# Intestinal dysbiosis exacerbates the pathogenesis of psoriasis-like phenotype through changes in fatty acid metabolism

**DOI:** 10.1038/s41392-022-01219-0

**Published:** 2023-01-30

**Authors:** Qixiang Zhao, Jiadong Yu, Hong Zhou, Xiaoyan Wang, Chen Zhang, Jing Hu, Yawen Hu, Huaping Zheng, Fanlian Zeng, Chengcheng Yue, Linna Gu, Zhen Wang, Fulei Zhao, Pei Zhou, Haozhou Zhang, Nongyu Huang, Wenling Wu, Yifan Zhou, Jiong Li

**Affiliations:** State Key Laboratory of Biotherapy and Cancer Center, West China Hospital, West China Medical School, Sichuan University, and Collaborative Innovation Center for Biotherapy, Chengdu, China

**Keywords:** Immunological disorders, Adaptive immunity, Microbiology, Bioinformatics

## Abstract

The intestinal microbiota has been associated with host immunity as well as psoriasis; however, the mechanism of intestinal microbiota regulating psoriasis needs to be demonstrated systematically. Here, we sought to examine its role and mechanism of action in the pathogenesis of psoriasis. We found that the severity of psoriasis-like skin phenotype was accompanied by changes in the composition of the intestinal microbiota. We performed co-housing and fecal microbial transplantation (FMT) experiments using the K14-VEGF transgenic mouse model of psoriasis and demonstrated that the transfer of intestinal microbiota from mice with severe psoriasis-like skin phenotype exacerbated psoriasiform skin inflammation in mice with mild symptoms, including increasing the infiltration and differentiation of Th17, and increased the abundance of *Prevotella*, while decreasing that of *Parabacteroides distasonis*, in the colon. These alterations affected fatty acid metabolism, increasing the abundance of oleic and stearic acids. Meanwhile, gentamicin treatment significantly reduced the abundance of *Prevotella* and alleviated the psoriasis-like symptoms in both K14-VEGF mice and imiquimod (IMQ)-induced psoriasis-like mice. Indeed, administration of oleic and stearic acids exacerbated psoriasis-like symptoms and increased Th17 and monocyte-derived dendritic cell infiltration in the skin lesion areas in vivo, as well as increased the secretion of IL-23 by stimulating DCs in vitro. At last, we found that, treatment of PDE-4 inhibitor alleviated psoriasis-like phenotype of K14-VEGF mice accompanied by the recovery of intestinal microbiota, including the decrease of *Prevotella* and increase of *Parabacteroides distasonis*. Overall, our findings reveal that the intestinal microbiota modulates host metabolism and psoriasis-like skin inflammation in mice, suggesting a new target for the clinical diagnosis and treatment of psoriasis.

## Introduction

Psoriasis is a skin-specific, immune-mediated disease that seriously endangers human health.^1^ It affects approximately 2% of the population in Europe and North America, and there are more than 6 million psoriasis patients in China.^2,3^ The pathogenesis is mainly mediated by dendritic cells (DCs), T cells, and keratinocytes.^4^ Among them, Th17 cells play an important role in the occurrence and development of psoriasis by producing IL-17A, which stimulates several responses that promote keratinocyte hyperproliferation, epidermal hyperplasia, and skin inflammation.^5,6^

Intestinal microorganisms are important factors affecting host health which influence the pathogenesis of immune-related diseases by regulating innate and adaptive immunity mediated by DCs, Th17 cells, epithelial cells, and goblet cells.^7,8^ *Candidatus Arthromitus* was reported to increase in the intestines of mice with autoimmune arthritis and it activated the differentiation of Th17 cells as well as induced autoimmune arthritis by promoting the secretion of serum amyloid A.^9,10^ Several studies examining differences in the composition of the intestinal microbiota between psoriasis patients and healthy controls have suggested that disorders of the intestinal microbiota may be closely related to the development of psoriasis.^11–13^ Germ-free (GF) mice develop more severe psoriasis-like skin inflammation than conventional mice due to enhanced Th17 responses, indicating that the intestinal microbiota may indeed be crucial in the progression of psoriasis.^14^ Moreover, sPLA2-IIA was contributed to the shaping of the gut microbiota and Pla2g2a^–/–^ mice showed an altered intestinal microbiota which regulated the pathogenesis of psoriasis and skin carcinogenesis through co-housing experiment.^15^

However, a systematic study is needed to demonstrate the relationship between intestinal microbe and psoriasis, including finding the microbes that promote or inhibit psoriasis, exploring the mechanism of intestinal microbes regulating psoriasis, and expounding its effect on host immunity. We observed that if mice with a mild pre-psoriatic phenotype are co-housed with severely psoriatic mice, their psoriatic pathogenesis is aggravated. Based on this observation and the recent studies linking the intestinal microbiota to psoriasis, we hypothesized that the development of psoriasis is mediated by the intestinal microbiota.

Herein, we sought to examine this hypothesis. To that aim, we performed co-housing and fecal microbial transplantation (FMT) experiments using K14-VEGF transgenic mice, which develop an inflammatory condition resembling human psoriasis and are widely used as a model for studying psoriasis.^16,17^ We found that the intestinal microbiota of mice with a dramatic psoriasiform phenotype aggravated the psoriasis-like phenotype of mild pre-psoriatic mice. Reversely, psoriasis-like mice were treated with antibiotics to reduce the intestinal microorganism that promoted psoriasiform skin inflammation and our results showed that this intervention alleviated the pathogenesis of psoriasis-like skin phenotype. Furthermore, we demonstrated that the transfer of intestinal microbiota changed the composition of intestinal metabolites, and that increases in oleic acid and stearic acid content exacerbated the psoriatic phenotype through their effect on Th17 cells and DCs. In the end, we found that therapy and alleviation of psoriasis-like skin phenotype led to the recovery of intestinal microbiota.

## Results

### Severity of psoriasis-like skin phenotype increases with age in K14-VEGF transgenic mice, and this is accompanied by changes in the composition of the intestinal microbiota

**Fig. 1:**
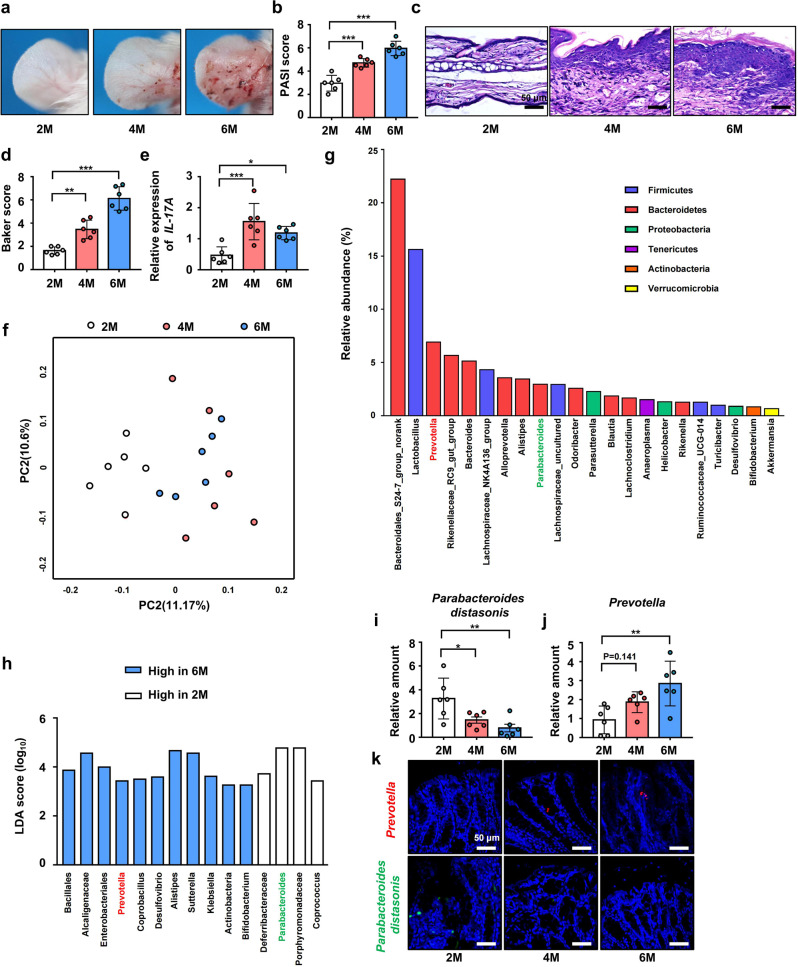
Severity of psoriasis-like skin phenotype increases with age in K14-VEGF transgenic mice, and this is accompanied with changes in the composition of the intestinal microbiota. **a** Macroscopic characteristics of the ears in 2 M, 4 M, and 6 M mice. **b** PASI score of ears in 2 M, 4 M, and 6 M mice. **c** Representative H&E staining of ears of 2 M, 4 M, and 6 M mice. (Scale bars: 50 μm). **d** Pathological score of ear sections using the Baker scoring system. **e** Relative mRNA expression of *IL-17A* in the ears of 2 M, 4 M, and 6 M mice. **f** Principal coordinates analysis (PcoA) of unweighted UniFrac distance based on 16s rDNA profiling of feces from 2 M, 4 M, and 6 M mice. **g** Relative abundance of dominant species in the feces from 2 M, 4 M, and 6 M mice. **h** Results of linear discriminant analysis effect size (LEfSe) analysis showing bacteria, at the lowest taxonomic level, that were significantly different in abundance in 2 M and 6 M mice. **i**, **j** Fecal contents of 2 M, 4 M, and 6 M mice were analyzed for *Prevotella* and *Parabacteroides distasonis* colonization by qPCR. **k** Representative fluorescence in situ hybridization for *Prevotella* (Prv392) and *Parabacteroides distasonis* (PD) in the colon. Data presented as mean ± SD on relevant graphs. *P ≤ 0.05; **P ≤ 0.01; ***P ≤ 0.005 (one-way ANOVA). 2 M (*n* = 6), 4 M (*n* = 6), and 6 M (*n* = 6). LDA linear discriminant analysis

First, we found that 6-month-old K14-VEGF (6 M) mice and 4-month-old K14-VEGF (4 M) mice had significantly more severe psoriasis-like phenotype than 2-month-old (2 M) mice (Fig. 1a). In addition, the Psoriasis Area and Severity Index (PASI) score of 6 M and 4 M mice was higher than that of 2 M mice (Fig. 1b). Histological examination of ear sections stained with hematoxylin and eosin (H&E) revealed the severity of the psoriasis-like symptoms in 2 M, 4 M, and 6 M mice (Fig. 1c). Based on the Baker scoring system, 6 M mice and 4 M mice exhibited higher scores (Fig. 1d) and thicker epidermis (Supplementary Fig. 1a) than 2 M mice. Furthermore, 6 M and 4 M mice had higher *IL-17A* mRNA levels in the ears than 2 M mice (Fig. 1e). IL-17 protein was detected by western blot and the result showed that 6 M and 4 M mice had higher IL-17A protein levels in the ears than 2 M mice (Supplementary Fig. 1b, c).

Second, we found that the increase in the severity of the psoriasis-like phenotype was accompanied by differences in the composition of the intestinal microbiota among 2 M, 4 M, and 6 M mice (Fig. 1f, Supplementary Fig. 1g). By analyzing the dominant species in the feces from 2 M, 4 M, and 6 M mice (Fig. 1g), we found that, the abundance of *Prevotella* was higher in 6 M mice, and *Parabacteroides distasonis* was higher in 2 M mice (Fig. 1h). These results were verified by qPCR (Fig. 1i, j). In addition, the abundance of *Alistipes* was higher in 6 M mice (also increased in mice after co-housing and FMT) and *Alistipes* was reported to be more abundant in patients after the higher-fat diet intervention, which indicated its potential role in abnormal lipid metabolism.^18^ To detect the colonization of *Parabacteroides distasonis* and *Prevotella* in colon, bacterial FISH (Fluorescence in Situ Hybridization) was performed and the result showed that colonic sections from 2 M, 4 M, and 6 M mice exhibited increased hybridization of the *Prevotella* probe Prv392^19^ and decreased hybridization of the *Parabacteroides distasonis* probe PD (Fig. 1k).^20^

The number of colonic goblet cells and mucus content are correlated with the colonization rate of some microorganisms. For example, colonization by *Akkermansia muciniphila* increases the thickness of the intestinal mucus layer,^21,22^ and its abundance is positively correlated with the number of goblet cells and the expression of mucin 2 (Muc-2).^23^ Similarly, *Prevotella* is an intestinal microbe that colonizes the mucus layer of mammals and uses mucus as a source of nitrogen and carbon.^24,25^ Here, histological staining with Alcian Blue (AB)-Periodic Acid Schiff (PAS) revealed that the number of colonic mucus-secreting goblet cells in 6 M mice was significantly higher than that in 2 M mice while the number of goblet cells in 4 M mice was slightly higher than that in 2 M mice (Supplementary Fig. 1d, e). In addition, although 4 M mice had slightly higher *Muc-2* mRNA levels in the colon than 2 M mice, 6 M mic had significantly higher *Muc-2* mRNA levels in the colon than 2 M mice. (Supplementary Fig. 1f). These results suggested that higher *Muc-2* expression in 6 M and 4 M mice may lead to increased colonization by *Prevotella*. It has been reported that aged mice exhibited a decrease in beneficial intestinal microbiota and an increase in proinflammatory intestinal microbiota.^26^ Aging of mice may lead to the disorder of the intestinal microbiome and aggravate psoriasiform skin inflammation. In our study, WT-2M, WT-4M, and WT-6M mice did not show the aggravation of psoriasis-like phenotype companies with age (Supplementary Fig. 1h). To verify this, we performed FISH and found that colonic sections from WT-2M, WT-4M and WT-6M mice also exhibited increased hybridization of the *Prevotella* probe Prv392 and decreased hybridization of the *Parabacteroides distasonis* probe PD (Supplementary Fig. 1i). However, the abundance of *Prevotella* in WT mice was lower than that in K14-VEGF mice in same age (Fig. 1k). In addition to aging, it was demonstrated that skin inflammation also contributed to the dysbiosis of intestinal microbiota.^27^ Therefore, changes in intestinal microbiota in K14-VEGF mice are contributed by both aging and inflammation. These results indicated that the severity of psoriasis-like skin phenotype increases with age in 2 M, 4 M, and 6 M mice, which was accompanied by changes in the composition of the intestinal microbiota and 6 M mice exhibited increased colonization of the *Prevotella* and decreased colonization of *Parabacteroides distasonis* in the colon.

### Co-housing with 6 M mice exacerbated psoriasis-like skin phenotype in and changed the intestinal microbiota composition of, 2 M mice

**Fig. 2:**
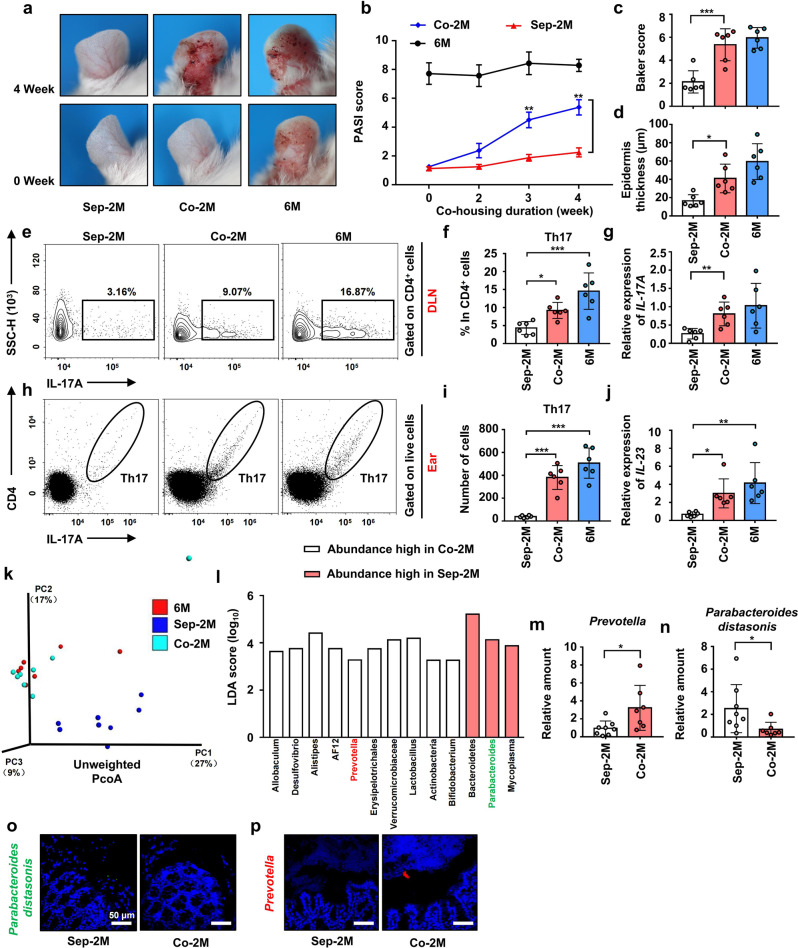
Co-housing with 6 M mice exacerbated psoriasis-like skin phenotype in and changed the intestinal microbiota composition of, 2 M mice. **a** Macroscopic characteristics of the ears of Sep-2M Co-2M and 6 M mice in 0 week and 4 weeks. **b** PASI score of ears in Sep-2M Co-2M and 6 M mice from 0 week to 4 weeks. **c** Pathological score of ear sections using the Baker scoring system. **d** Average epidermal thickness. **e** Analysis of Th17 cells (CD4^+^ IL-17^+^) by flow cytometry in DLNs. **f** Percentage of Th17 cells in CD4^+^ cells. **g** Relative mRNA expression of *IL-17A* in the ears. **h** Analysis of Th17 cells (CD4^+^ IL-17^+^) by flow cytometry in ears. **i** Number of Th17 cells in ears. **j** Relative mRNA expression of *IL-23* in the ears. **k** PcoA of unweighted UniFrac distance based on 16s rDNA profiling of feces from Sep-2M Co-2M and 6 M mice. **l** Results of LEfSe analysis showing bacteria, at the lowest taxonomic level, that were significantly different in abundance in Sep-2M and Co-2M mice. **m**, **n** Fecal contents of Sep-2M and Co-2M mice were analyzed for *Prevotella* and *Parabacteroides distasonis* colonization by qPCR. **o**, **p** Representative fluorescence in situ hybridization for *Prevotella* (Prv392) and *Parabacteroides distasonis* (PD) in the colon. Data presented as mean ± SD on relevant graphs. *P ≤ 0.05; **P ≤ 0.01; ***P ≤ 0.005. A two-tailed Student’s *t*-test was used in (**m**) and (**n**), one-way ANOVA was used for comparing the rest of the groups. (*n* = 6) in (**a**–**j**). Sep-2M (*n* = 8), Co-2M (*n* = 7) and 6 M (*n* = 7) in (**g**, **h**). (*n* = 6) in **o**, **p**

To explore the regulation effect of intestinal microbiota on psoriasis, we performed co-housing experiments and found that, compared with separately housed 2 M (Sep-2M) mice, co-housed 2 M (Co-2M, co-housed with 6 M mice) mice had more severe psoriasis-like skin phenotype after four weeks (Fig. 2a). The PASI score of Co-2M mice was already increased after co-housing for 2 weeks and significantly increased after three weeks compared with Sep-2M mice (Fig. 2b). After co-housing, ear sections were stained with H&E (Supplementary Fig. 2a). Co-2M mice showed a higher Baker score and thicker epidermis than Sep-2M mice (Fig. 2c, d). To explore whether infiltration of immune cells into the ears and the aggravated skin inflammation were related to Co-2M mice with exacerbated psoriasiform skin inflammation, flow cytometry was performed to detect the Th1, Th2, Th17, and Treg cells in the ears and Draining lymph nodes (DLNs). The result showed that only a few infiltrations of Th1, Th2, and Treg cells the ears were detected (Supplementary Fig. 2h, j). Besides, the differentiation of Th1, Th2, and Treg in DLNs showed no significant difference among the three groups (Supplementary Fig. 2i, k).

However, compared with Sep-2M mice, differentiation of Th17 cells was increased in DLNs of Co-2M mice and 6 M mice (Fig. 2e, f). Furthermore, infiltration of Th17 into the ears was also detected by flow cytometry (Fig. 2h), and the results demonstrated an increase in Co-2M mice and 6 M mice (Fig. 2i). Compared with Sep-2M mice, Co-2M mice and 6 M showed higher *IL-17A* and *IL-23* mRNA expressions in the ears (Fig. 2g, j). Meanwhile, IL-17A protein was significantly higher in the ears of Co-2M mice and 6 M mice (Supplementary Fig. 2b, c). We also detected the mRNA expressions of *IL-21* and *IL-22* and found that expressions of *IL-22* were slightly increased in Co-2M mice and 6 M mice while there was no significant difference in *IL-21* levels among the three groups (Supplementary Fig. 2d, e). These results suggested that the L-23-IL-17 immune axis is important in the aggravation of psoriasiform skin inflammation.

Keratin 10(KRT10) is a keratin marker produced in well-differentiated upper basal keratinocytes and was reported to be significantly decreased in lesions of psoriasis.^28,29^ We detected the mRNA expression of *KRT10* and found that the expression of *KRT10* was significantly decreased in the ears of Co-2M mice and 6 M mice compared with Sep-2M (Supplementary Fig. 2f). We also detected the expression of *S100A7* which is a proinflammatory protein belonging to the S100 family and is significantly overexpressed in psoriasis.^30^ The result showed that the mRNA expression of *S100A7* was significantly increased in the ears of Co-2M mice and 6 M mice compared with Sep-2M (Supplementary Fig. 2g).

Changes in the composition of the intestinal microbiota provided evidence for increased skin inflammation. 16 S rRNA gene sequencing showed that compared with Sep-2M mice, Co-2M mice exhibited an increase in total species diversity and observed species (Supplementary Fig. 3j, k), suggesting the transfer of intestinal microbiota. Furthermore, the operational taxonomic units (OTUs) in Co-2M mice were more similar to those in 6 M mice than those in Sep-2M mice (Supplementary Fig. 3l). In terms of β-diversity, Co-2M and 6 M mice showed similar intestinal microbiota composition, which was markedly distinct from that of Sep-2M mice (Fig. 2k). In addition, the abundance of *Prevotella* was increased, while that of *Parabacteroides distasonis* was decreased, in Co-2M mice after co-housing (Fig. 2l and Supplementary Fig. 3m). The qPCR results showed that the relative abundance of *Prevotella* was increased in the fecal samples of Co-2M mice, while that of *Parabacteroides distasonis* was decreased (Fig. 2m, n). Results of FISH showed that colonic sections from Co-2M mice exhibited an increased abundance of *Prevotella* and a decreased of *Parabacteroides distasonis* compared with Sep-2M mice (Fig. 2o, p). These results suggested that the intestinal microorganisms from mice with severe psoriasis-like symptoms, may exacerbate psoriasiform skin inflammation in mice with mild symptoms.

Disorders of the intestinal microbiota are related to intestinal inflammation.^31,32^ To explore the potential mechanisms whereby the intestinal microbiota may influence psoriasis, we studied the barrier, absorptive function, and inflammation of the intestines in mice. The length of the colon, histological score, and villus length in Co-2M and Sep-2M mice were not statistically significantly different (Supplementary Fig. 3a–d). Immunohistochemistry (IHC) was performed to examine the number of immune cells in the colon, and no significant differences in the number of CD45^+^ cells (immune cells) were found between in Co-2M and Sep-2M mice (Supplementary Fig. 3e, f). Moreover, Co-2M and Sep-2M mice showed no significant differences in the colonic mRNA expression of *IL-17A* (Supplementary Fig. 3g).

Intestinal microorganisms have been reported to prevent deterioration caused by hepatic injury and reduce neutrophil infiltration in the liver by enhancing the integrity of the intestinal epithelial barrier and increasing the expression of claudin-3, a tight junction integrin closely related to the integrity of the intestinal epithelium;^33,34^ therefore, IHC was performed to examine the colonic expression of claudin-3. No significant differences were found between Co-2M and Sep-2M mice (Supplementary Fig. 3h, i). Supplemental information above suggested that intestinal microbiota did not affect the pathogenesis of psoriasis by affecting the intestinal barrier and inflammation.

### Co-housing with 6 M mice changed the intestinal metabolite composition and metabolic pathways of 2 M mice

**Fig. 3:**
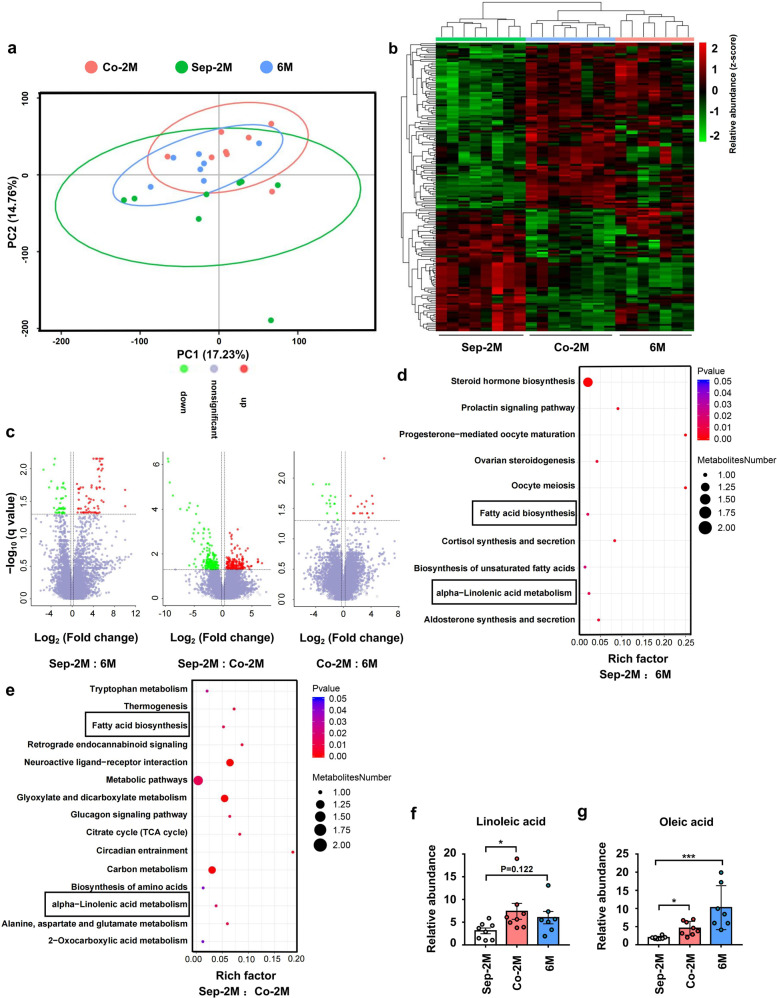
Co-housing with 6 M mice changed the intestinal metabolite composition and metabolic pathways of 2 M mice. **a** Principal components analysis (PCA) of fecal metabolites from Sep-2M Co-2M and 6 M mice. **b** Heatmap of metabolites identified in fecal samples. **c** Volcano plot of metabolites identified in fecal samples. Volcano plots are depicted with the fold change of each metabolite and the *q* value was calculated by performing a *t*-test and corrected by the false discovery rate. **d**, **e** Pathway enrich analysis of metabolites from Sep-2M and Sep-6M mice, and Sep-2M and Co-2M mice. **f**, **g** Relative abundance of linoleic acid and oleic acid in fecal metabolites. Data presented as mean ± SD on relevant graphs. *P ≤ 0.05; **P ≤ 0.01; ***P ≤ 0.005 (one-way ANOVA). Sep-2M (*n* = 8), Co-2M (*n* = 8), and 6 M (*n* = 7)

The intestinal microbiota was reported to regulate amino acid metabolism in the intestines, thereby inhibiting Alzheimer’s disease progression.^35^ Thus, we hypothesized that the intestinal metabolites resulting from microbial metabolism have an impact on psoriasis susceptibility. We utilized metabolomic profiling to identify candidate microbiota-dependent molecules in fecal samples of 6 M, Co-2M, and Sep-2M mice in the co-housing experiment as previously mentioned. (Supplementary Fig. 4a). After co-housing, the intestinal metabolite composition of Co-2M mice was similar to that of 6 M mice (Fig. 3a). A heatmap of metabolites and a volcano plot revealed similar metabolite abundance in Co-2M and 6 M mice (Fig. 3b, c). Pathway enrichment analysis showed differences in α-linolenic acid metabolism and fatty acid biosynthesis between Sep-2M and 6 M mice, and between Sep-2M and Co-2M mice (Fig. 3d, e). Moreover, the relative abundance of oleic acid was significantly higher in 6 M and Co-2M mice and the relative abundance of linoleic acid was significantly higher in Co-2M mice while slightly higher in 6 M mice compared with 2 M mice. (Fig. 3f, g), In addition, the relative abundance of traumatic acid was higher in Sep-2M mice Supplementary Fig. 4b). These data indicated that intestinal microbiota-derived changes in fatty acid metabolism may play a role in the development of psoriasis-like skin phenotype.

### FMT from 6 M to 2 M mice significantly exacerbated psoriasis-like skin phenotype and increased the infiltration of Th17 cells into the ear by changing the composition of intestinal microbiota

**Fig. 4:**
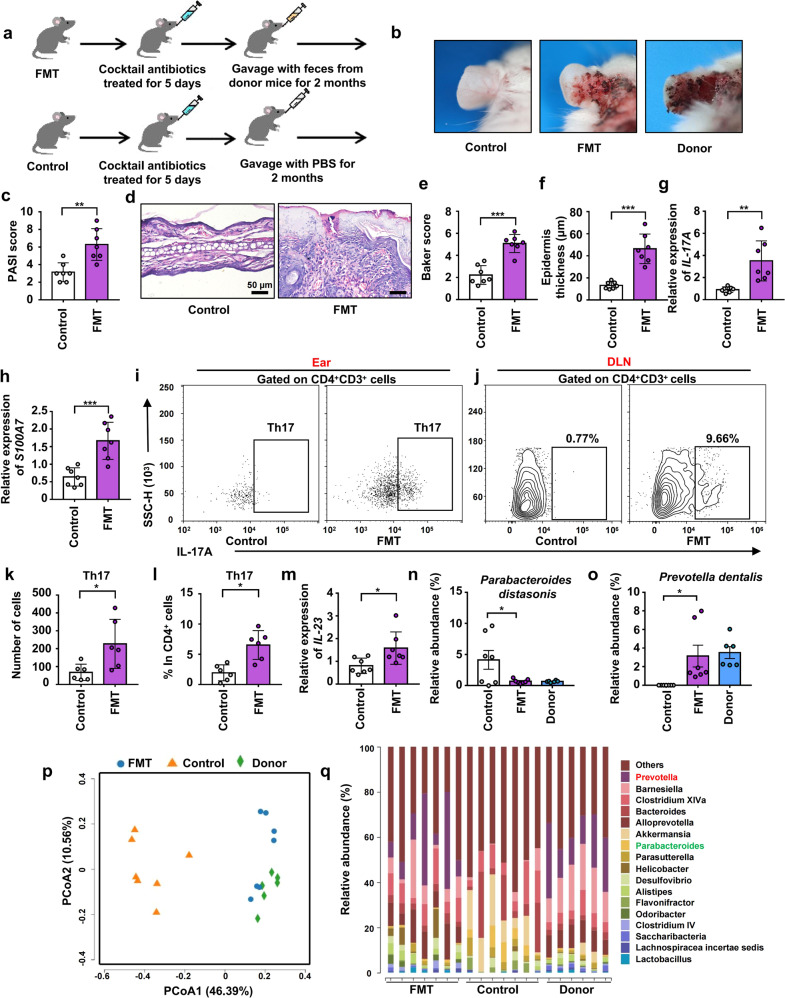
FMT from 6 M to 2 M mice significantly exacerbated psoriasis-like skin phenotype and increased the infiltration of Th17 cells into the ear by changing the composition of intestinal microbiota. **a** Scheme of the experimental design. **b** Macroscopic characteristics of the ears in FMT, Control and Donor mice. **c** PASI score of ears. **d** Representative H&E staining of ears (Scale bars: 50 μm). **e** Baker scoring. **f** Average epidermal thickness. **g**, **h** Relative mRNA expression of *IL-17A* and *S100A7* in ears. **i**, **j** Analysis of Th17 cells (CD4^+^ IL-17^+^) by flow cytometry in ears and DLNs. **k** Number of Th17 cells in ears. **l** Percentage of Th17 cells in CD4^+^ cells. **m** Relative mRNA expression of *IL-23* in ears. **n**, **o** Relative abundance (%) of *Parabacteroides distasonis* and *Prevotella dentalis* in the Fecal contents of FMT, Control and Donor mice. **p** PcoA of unweighted UniFrac distance. **q** Taxonomic distributions of bacteria. Data presented as mean ± SD on relevant graphs. *P ≤ 0.05; **P ≤ 0.01; ***P ≤ 0.005; Two-tailed Student’s *t*-test was used in (**c**–**m**) and one-way ANOVA was used in (**n**, **o**). (*n* = 7) in (**c**–**h**, **m**). (*n* = 6) in (**i**–**l**). Control (*n* = 7), FMT (*n* = 7) and Donor (*n* = 6) in (**n**–**q**)

FMT is an effective method for intestinal microbial colonization.^21,35^ To certain that microbes from the intestine regulated the progression of psoriasis, FMT was performed. Here, we divided 2 M mice into transplanted and control groups, and treated them with a cocktail of antibiotics to reduce their intestinal microbiota. Then, mice in the transplanted group were gavaged with a fecal microbiota filtrate from 6 M mice (donor mice), while control mice were gavaged with reduced PBS instead (Fig. 4a). After colonization for 2 months, the severity of psoriasiform skin inflammation in the transplanted group was significantly higher than that in the control group (Fig. 4b). In addition, the PASI score of transplanted mice was significantly higher than that of control mice (Fig. 4c). After FMT, mice were sacrificed and ear sections were stained with H&E (Fig. 4d). Histological photographs of the ears showed higher Baker score and thicker epidermis in the transplanted group than in the control group (Fig. 4e, f). mRNA expression of *IL-17A*, *S100A7* and *IL-23* were increased while *KRT10* was decreased in the ears of the transplanted group (Fig. 4g, h, m and Supplementary Fig. 5l). Furthermore, we examined the infiltration of Th17 cells in the ears and differentiation of Th17 cells in DLNs using flow cytometry (Fig. 4i, j). Transplanted mice showed more infiltration of Th17 cells in the ears and more differentiation of Th17 cells in the DLNs than control mice (Fig. 4k, l). However, there were still no significant differences between transplanted and control mice in terms of immune cell infiltration in the colonic lamina propria (Supplementary Fig. 5j, k).

After antibiotic treatment, the number of species detected in the intestines of mice in the transplanted and control groups decreased significantly (Supplementary Fig. 5a, b). After FMT, species abundance and diversity in transplanted mice were significantly higher than those in control mice (Supplementary Fig. 5c, d). Moreover, transplanted mice shared more OTUs with donor mice than with control mice (Supplementary Fig. 5e). The intestinal microbial composition of transplanted mice was remarkably similar to that of donor mice and significantly different from that of control mice (Fig. 4p). The abundance of *Parabacteroides distasonis* was decreased while *Prevotella dentalis* was increased after transplantation (Fig. 4n, o, q). Furthermore, the abundances of *Bacteroides acidifaciens*, *Alloprevotella rava, Alistipes onderdonkii*, and *Saccharibacteria* were increased in transplanted mice (Fig. 4q and Supplementary Fig. 5f–i). Taken together, these results strongly suggested that intestinal microbiota transplanted from 6 M to 2 M mice significantly increased the abundance of *Prevotella dentalis* as well as decreased the abundance of *Parabacteroides distasonis* and thus exacerbated psoriasis-like skin phenotype and increased the infiltration of Th17 cells into the ear.

### Fecal microbial transplantation from donor mice altered the intestinal metabolic composition and metabolic pathways of transplanted mice

**Fig. 5:**
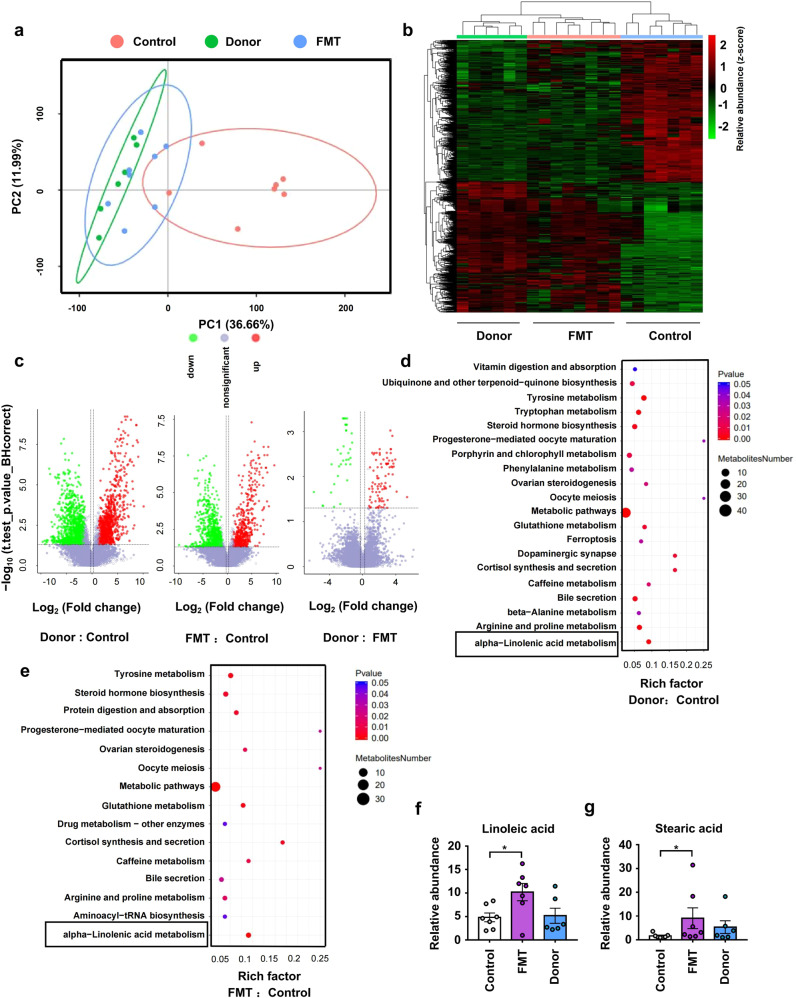
Fecal microbial transplantation from donor mice altered the intestinal metabolic composition and metabolic pathways of transplanted mice. **a** PCA of fecal metabolites from FMT, Control, and Donor mice. **b** Heatmap of metabolites identified in fecal samples. **c** Volcano plot of metabolites identified in fecal samples. Volcano plots are depicted with the fold change of each metabolite and the *q* value was calculated by performing a *t*-test and corrected by the false discovery rate. **d**, **e** Pathway enrich analysis of metabolites from Donor and Control mice, and FMT and Control mice. **f**, **g** Relative abundance of linoleic acid and stearic acid in fecal metabolites. Data presented as mean ± SD on relevant graphs. *P ≤ 0.05; **P ≤ 0.01; ***P ≤ 0.005 (one-way ANOVA). Control (*n* = 7), FMT (*n* = 7), and Donor (*n* = 6)

To examine whether the intestinal microbiota from psoriatic mice mediated the aggravation of psoriasis by regulating intestinal fatty acid metabolism, we analyzed the intestinal metabolism of mice in the FMT experiment as previously mentioned. (Supplementary Fig. 4d). The intestinal metabolite composition of transplanted mice was similar to that of donor mice and different from that of control mice (Fig. 5a). A heatmap of metabolites and a volcano plot revealed similar metabolite abundance in FMT and Donor mice (Fig. 5b, c). In addition, similar to the results of the co-housing experiments, the α-linoleic acid metabolism pathway was different between donor and control mice as well as between transplanted and control mice (Fig. 5d, e). The abundance of stearic acid and linoleic acid was higher in transplanted mice (Fig. 5f, g), while that of traumatic acid was higher in control mice (Supplementary Fig. 4c). These results also indicated that intestinal microbiota-derived changes in fatty acid metabolism may regulate the psoriasis-like skin phenotype.

### Administration of gentamicin alleviated the pathogenesis of psoriasis-like skin phenotype in K14-VEGF mice with the decreased infiltration of Th17

**Fig. 6:**
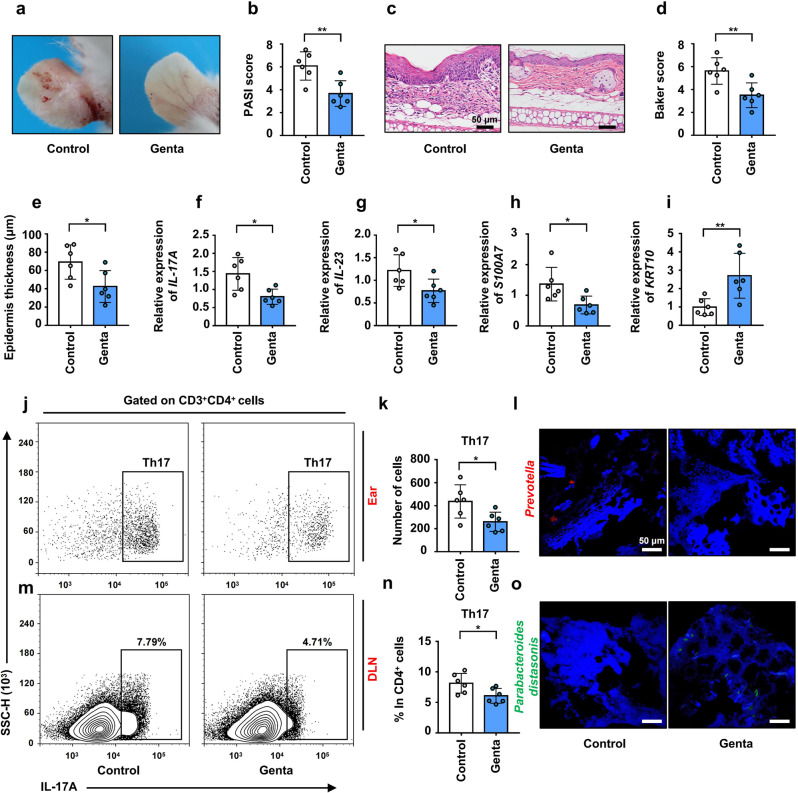
Administration of gentamicin alleviated the pathogenesis of psoriasis-like skin phenotype in K14-VEGF mice with the decreased infiltration of Th17. **a** Macroscopic characteristics of the ears in Control and Genta group mice 4–5 M (18 weeks). **b** PASI score of ears in Control and Genta group mice. **c** Representative H&E staining of ears (Scale bars: 50 μm). **d** Pathological score of ear sections using the Baker scoring system. **e** Average epidermal thickness. **f**–**i** Relative mRNA expression of *IL-17A*, *IL-23*, *S100A7*, and *KRT10* in the ears of Control and Genta group mice. **j** Analysis of Th17 cells (CD4^+^ IL-17^+^) by flow cytometry in ears. **k** Number of Th17 cells in ears. **l** Representative fluorescence in situ hybridization for *Prevotella* (Prv392) in colonic contents. **m** Analysis of Th17 cells (CD4^+^ IL-17^+^) by flow cytometry in DLNs. **n** Percentage of Th17 cells in CD4^+^ cells. **o** Representative fluorescence in situ hybridization for *Parabacteroides distasonis* (PD) in colonic contents. Data presented as mean ± SD on relevant graphs. *P ≤ 0.05; **P ≤ 0.01; ***P ≤ 0.005 (Two-tailed Student’s *t*-test). (*n* = 6)

To verify that increased *Prevotella* promoted the aggravation of psoriasis, K14-VEGF mice was treated with antibiotics to reduce the abundance of *Prevotella*. Four to 5 months (18 weeks) mice were chosen to explore the regulation of antibiotics on psoriasis. It was demonstrated that *Prevotella* is highly sensitive to gentamicin and the abundance of *Prevotella* was significantly decreased in mice treated with gentamicin for 6 days.^36^ After administration of gentamicin for 15 days, *Prevotella* in colonic contents was detected by FISH in K14-VEGF mice and the result showed that the abundance of *Prevotella* was significantly decreased in the gentamicin group (Fig. 6l). The severity of psoriasis-like skin phenotype in the gentamicin group was significantly lower than that in the control group (Fig. 6a). In addition, the PASI score of gentamicin-treated mice was significantly lower than that of control mice (Fig. 6b). After administration of gentamicin, mice were sacrificed and ear sections were stained with H&E (Fig. 6c). Histological photographs of the ears showed lower Baker score and thinner epidermis in the gentamicin group than in the control group (Fig. 6d, e). Furthermore, we examined the infiltration of Th17 cells in ears and the differentiation of Th17 cells in DLNs using flow cytometry (Fig. 6j, m). Gentamicin-treated mice showed less infiltration of Th17 cells in the ears and less differentiation of Th17 cells in the DLNs than control mice (Fig. 6k, n). In addition, we detected the expression of related cytokines in the ears of K14-VEGF mice and found that the mRNA expression of *IL-17A*, *IL-23*, and *S100A7* were decreased while *KRT10* was increased after the administration of gentamicin (Fig. 6f–i). Besides, we detected the abundance of *Parabacteroides distasonis* in the colonic contents of K14-VEGF mice and found it increased after administration of gentamicin which is consistent with our previous results (abundance of *Parabacteroides distasonis* and *Prevotella* is inversely correlated) (Fig. 6o). This result is also consistent with the research result of Hesuiyuan Wang and his colleagues who demonstrated that *Parabacteroides distasonis* was significantly increased after administration of gentamicin.^37^ In general, administration of gentamicin alleviated the pathogenesis of psoriasis-like skin phenotype in K14-VEGF mice by reducing the abundance of *Prevotella* and increasing the abundance of *Parabacteroides distasonis*.

In addition, we also verified this in the imiquimod (IMQ)-induced psoriasis-like skin mouse model. After administration of gentamicin for 10 days (twice a day), mice were treated with IMQ for 5 days. During the treatment of IMQ, gentamicin group mice were still gavaged with gentamicin (once a day). After administration of gentamicin for 15 days, an abundance of *Prevotella* was decreased while the abundance of *Parabacteroides distasonis* was increased in the colonic contents of mice (Supplementary Fig. 6l, n). Our results showed that administration of gentamicin alleviated pathogenesis of psoriasis-like symptoms, including alleviating psoriasiform skin inflammation, a decrease in the skin epidermis thickness, decreasing mRNA expression of *IL-17A*, *IL-23*, *S100A7*, and increased mRNA expression of *KRT10* (Supplementary Fig. 6a–i). What’s more, gentamicin group mice showed a less infiltration of Th17 cells in the skin lesion area and less differentiation of Th17 cells in the DLN than control group mice (Supplementary Fig. 6j, k, m). However, gentamicin was less effective in alleviating psoriasiform skin inflammation in IMQ psoriasis-like skin mouse model than in the K14-VEGF mouse model, probably due to the higher colonization of *Prevotella* and *Parabacteroides distasonis* in the colon of K14-VEGF mice than IMQ-induced mice. In summary, these results indicated that administration of gentamicin depleted the *Prevotella* and thus alleviated the pathogenesis of psoriasis-like skin phenotype in K14-VEGF mice and IMQ-induced mice with the decreased infiltration of Th17.

### Administration of free fatty acid exacerbated the pathogenesis of psoriasis-like skin phenotype of 2 M K14-VEGF mice and promoted the differentiation of Th17 in DLN as well as increased the infiltration of monocyte-derived DCs and Th17 in ears

**Fig. 7:**
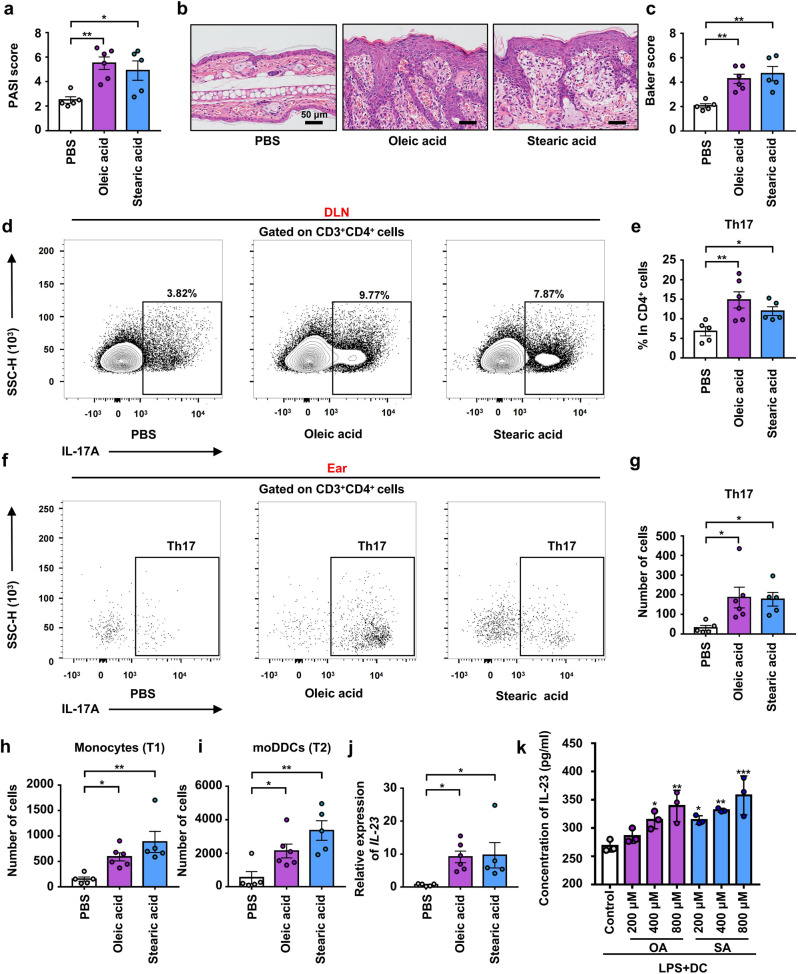
Administration of free fatty acid exacerbated the pathogenesis of psoriasis-like skin phenotype of 2 M K14-VEGF mice and promoted the differentiation of Th17 in DLN as well as increased the infiltration of moDDCs and Th17 in ears. **a** PASI score of ears in PBS, oleic acid, and stearic acid group mice. **b** Representative H&E staining of ears (Scale bars: 50 μm). **c** Pathological score of ear sections using the Baker scoring system. **d** Analysis of Th17 cells (CD4^+^ IL-17^+^) by flow cytometry in DLNs. **e** Percentage of Th17 cells in CD4^+^ cells. **f** Analysis of Th17 cells (CD4^+^ IL-17^+^) by flow cytometry in ears. **g** Number of Th17 cells in ears. **h** Number of Monocytes in ears. **i** Number of moDDCs in ears. **j** Relative mRNA expression of *IL-23* in the ears of PBS, oleic acid, and stearic acid group mice. **k** L-23 concentrations in the culture supernatants were measured by ELISA. Data presented as mean ± SD on relevant graphs. *P ≤ 0.05; **P ≤ 0.01; ***P ≤ 0.005 (one-way ANOVA). PBS (*n* = 5), oleic acid (*n* = 6), and stearic acid (*n* = 5) in (**a**–**j**) and (*n* = 3) in (**k**). OA oleic acid and SA stearic acid

Oleic acid and palmitic acid were reported to activate DCs, stimulate IL-23 secretion, enhance Th17 cell responses, and increase IL-17 expression in vitro.^38^ To explore the role of fatty acids in the regulation of psoriasis, 2 M mice were administered with either oleic and stearic acids (treated group) or PBS (control group). After 41 days of gavage, we found that oleic and stearic acid administration aggravated psoriasis-like symptoms, as evidenced by the swelling and scaling of the ear (Supplementary Fig. 7a). The treated group had a significantly higher PASI score than the control group (Fig. 7a). The ear was stained with H&E (Fig. 7b) and the Baker score showed that the treated group had significantly more severe psoriasis-like skin phenotype than control mice (Fig. 7c) and that the epidermis was significantly thickened (Supplementary Fig. 7b).

Flow cytometry was performed to examine Th17 cell differentiation in DLNs of the three groups (Fig. 7d). The results showed that, compared with control mice, Th17 cell differentiation was increased in the treated group (Fig. 7e). Furthermore, Th17 cell infiltration into the ear was also detected by flow cytometry (Fig. 7f), and the results demonstrated an increase in the treated groups (Fig. 7g). What’s more, an increase mRNA expression of *S100A7* and a decreased mRNA expression of *KRT10* were founded after the administration of oleic acid and stearic acid (Supplementary Fig. 7c, d).

IL-23 production by DCs is required for the differentiation, expansion, and survival of pathogenic Th17 cells.^39–41^ Therefore, we examined DC infiltration (Supplementary Fig. 7f). The results showed that the numbers of monocytes and monocyte-derived DCs (moDDCs) were significantly increased in the ears of treated mice (Fig. 7h, i and Supplementary Fig. 7f). MoDDCs were reported to mediate IMQ-induced psoriasis-like keratinocyte proliferation, thickening of the epidermis, and dermal inflammation.^15^ In addition, the mRNA expression of *IL-23* was increased in treated mice (Fig. 7j). In order to explore the regulation of oleic acid and stearic acid on DCs, we performed oleic acid and stearic acid stimulation experiment on DCs in vitro. MoDDCs were stimulated with 200–800 μM bovine serum albumin (BSA)-bound oleic acid (OA) or stearic acid (SA) while control MoDDCs were stimulated with BSA diluted in the solvent alone.^38^ Result of ELISA showed that, stimulating with oleic acid and stearic acid increased secretion of IL-23 in moDDCs (Fig. 7k).

In, addition, we also verified this in IMQ psoriasis-like skin mouse model. C57 mice were gavaged with oleic acid, stearic acid, or PBS for 41 days and were treated with IMQ in the last 5 days. Our results showed that administration of oleic acid and stearic acid aggravated the pathogenesis of psoriasis-like skin phenotype, including the exacerbating symptoms (Supplementary Fig. 8a, b), an increase in the skin inflammation and epidermis thickness (Supplementary Fig. 8c–e), increase mRNA expression of *IL-17A*, *IL-23*, *S100A7*, and decrease mRNA expression of *KRT10* in the skin lesion area (Supplementary Fig. 8i–l). Meanwhile, treated mice showed more infiltration of Th17 cells in the skin lesion area and more differentiation of Th17 cells in the DLNs than control group mice (Supplementary Fig. 8f–h). What’s more, treated mice showed more infiltration of moDDCs and monocytes in the skin lesion area than the control group mice (Supplementary Fig. 8m–p). Together, our results suggested that intestinal microbiota-derived increases in oleic and stearic acid levels sensitized DCs, increasing IL-23 expression and Th17 infiltration into the skin lesion area, thus exacerbating the pathogenesis of psoriasis-like skin phenotype in K14-VEGF and IMQ-induced mice (Fig. 9).

### Administration of PDE-4 inhibitor significantly improved psoriasis-like skin phenotype in mice and was accompanied by recovery of intestinal microbiota

**Fig. 8:**
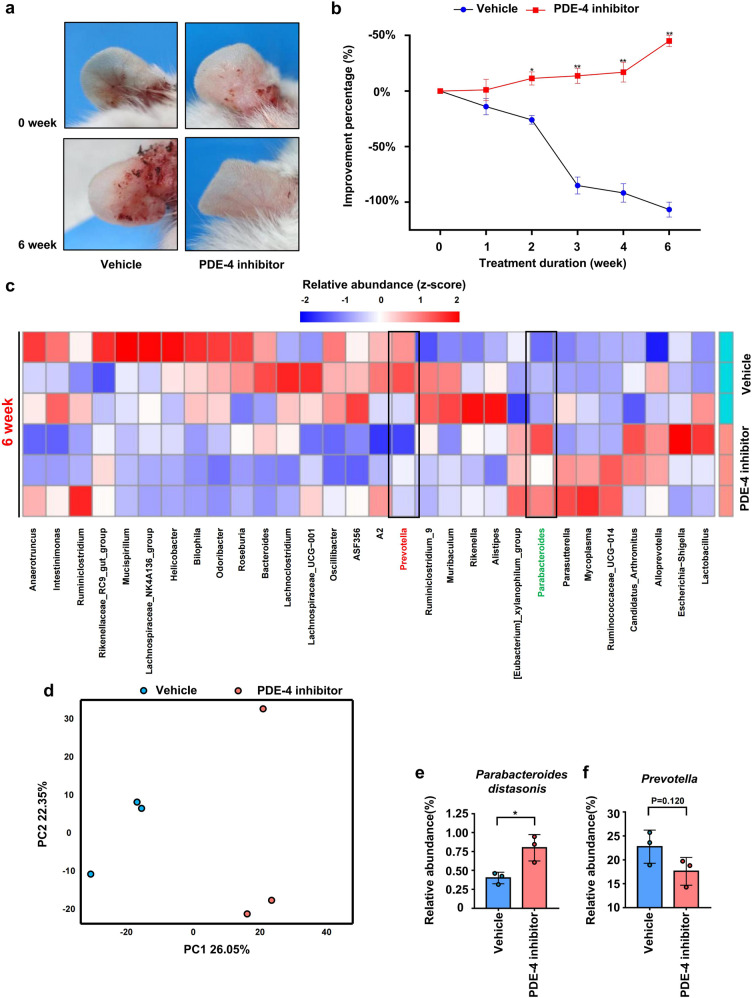
The administration of PDE-4 inhibitors significantly improved psoriasis-like skin phenotype in K14-VEGF mice and was accompanied by recovery of intestinal microbiota. **a** Macroscopic characteristics of the ears in Vehicle and PDE-4 inhibitor group mice in 0 week and 6 weeks. **b** Improvement percentages of PASI score of ears in Vehicle and PDE-4 inhibitor group mice from 0 week to 6 weeks. **c** Taxonomic distributions of bacteria. **d** PcoA of unweighted UniFrac distance based on 16s rDNA profiling of feces from Vehicle and PDE-4 inhibitor group mice. **e**, **f** Relative abundance *of Parabacteroides distasonis* and *Prevotella* in the feces of Vehicle and PDE-4 inhibitor group mice. Data presented as mean ± SD on relevant graphs. *P ≤ 0.05; **P ≤ 0.01; ***P ≤ 0.005 (two-tailed Student’s *t*-test) (*n* = 3)

Our above study showed that the aggravation of the psoriasis-like skin phenotype led to the disorder of intestinal microbiota; Disordered intestinal microbiota exacerbated the psoriasiform skin inflammation (Co-housing and FMT); Remodeling of intestinal microbiota relieved the psoriasis-like symptom (Gentamicin treated). Apremilast is a kind of PDE-4 inhibitor which was reported to specifically regulate the expression of proinflammatory and anti-inflammatory mediators in psoriasis and it was approved by FDA for the treatment of moderate-to-severe chronic plaque psoriasis.^42^ To investigate whether alleviation of psoriasis-like phenotype is accompanied by the recovery of intestinal microbiota, K14-VEGF mice (4 M) were treated with apremilast. The result showed that, apremilast significantly improved psoriasis-like phenotype in K14-VEGF mice after 6 weeks of treatment (Fig. 8a). The improvement percentage (according to the change of PASI score) of PDE-4 inhibitor-treated mice was significantly increased after two weeks compared with control mice (Fig. 8b). Feces were collected from both groups before treatment and 6 weeks after treatment. Although the abundance of *Parabacteroides distasonis* and *Prevotella* showed no significant differences between 0 and 6 weeks in the feces of PDE-4 inhibitor-treated mice (Supplementary Fig. 9b), the *Parabacteroides distasonis* was decreased while *Prevotella* was increased in 6-week samples compared with 0-week samples collected from control mice (Supplementary Fig. 9a) which is consistent with results of the previous horizontal comparison between groups.

**Fig. 9:**
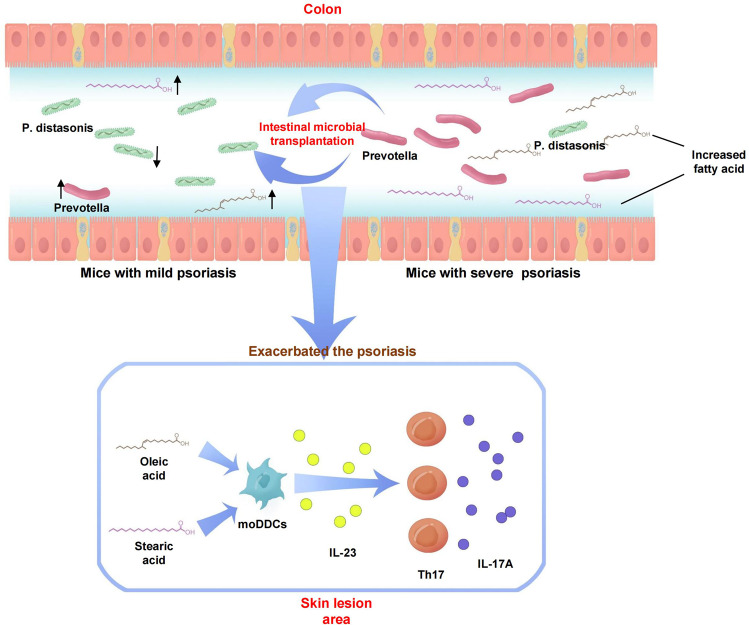
Schematic representation of the relationship between intestinal microbiota, fatty acid, and pathogenesis of psoriasis

We analyzed the intestinal microbiota composition of PDE-4 inhibitor-treated mice and control mice after administration for 6 weeks and found that the intestinal microbial composition of PDE-4 inhibitor-treated mice was remarkably separated from that of control mice (Fig. 8c). In addition, the abundance of *Parabacteroides distasonis* was significantly increased and abundance of *Prevotella* was slightly decreased in the feces of PDE-4 inhibitor-treated mice (Fig. 8d–f). The above results indicated that, although PDE-4 inhibitors did not directly restore the intestinal microbiota in psoriatic mice, they moderated its disorder. Besides, compared with control mice, PDE-4 inhibitor-treated mice showed an alleviative psoriasis-like phenotype accompanied by the recovery of intestinal microbiota.

## Discussion

Psoriasis is a skin-specific, immune-mediated disease that is mainly mediated by DCs, Th17 cells, and keratinocytes. Several studies have suggested that intestinal dysbiosis may be closely related to the development of immune-mediated diseases. However, the mechanism whereby the intestinal microbiota may mediate psoriasis progression remains to be studied. Therefore, we sought to investigate the role of the intestinal microbiota and its mechanism of action in the pathogenesis of psoriasis. To these aims, we employed the widely used K14-VEGF transgenic mouse model of psoriasis, and performed co-housing and fecal microbial transplantation experiments.

In examining the aspects mentioned above, we found that the severity of psoriasis-like skin phenotype in K14-VEGF mice is accompanied by changes in the intestinal microbiota. *Prevotella* abundance was high in the intestines of mice with severe psoriasis-like phenotype, while the abundance of *Parabacteroides distasonis* was high in mice with mild psoriasis-like phenotype. In addition, we found that co-housing and FMT exacerbate psoriasis-like symptoms in mice with mild psoriasiform skin inflammation, including ear inflammation, IL-17 expression, and Th17 infiltration as well as differentiation. Moreover, this was associated with an increase in *Prevotella* abundance and a decline in the abundance of *Parabacteroides distasonis* in the colon. Mice with severe psoriasis-like phenotype showed high mRNA expression of *Muc*-2 in the colon as well as increased numbers of mucus-secreting goblet cells suggesting the reason for the changes in intestinal microbiota. Furthermore, the changes in the intestinal microbiota disturbed the metabolic composition of the colon because fatty acid metabolism, including the α-linolenic acid metabolic pathway, was altered. Specifically, we found that the changes in the microbiota resulted in an increase in the abundance of colonic oleic, linoleic, and stearic acids, and a decrease in the abundance of traumatic acid. In turn, these changes aggravated psoriasis-like phenotype in K14-VEGF mice. To determine the role of intestinal microbiota in the pathogenesis of psoriasis, gentamicin was used to reduce the abundance of *Prevotella*. This intervention alleviated the pathogenesis of psoriasis-like symptoms in K14-VEGF mice and IMQ-treat mice, including skin inflammation, Th17 infiltration in skin, and Th17 differentiation in DLNs by reducing the abundance of *Prevotella* and increasing the abundance of *Parabacteroides distasonis*. To confirm the role of fatty acid metabolism disturbance in the progression of psoriasis, we administered oleic and stearic acids to K14-VEGF mice and IMQ-treat mice, and found that this aggravated psoriasiform skin inflammation by increasing Th17 cell differentiation in DLNs as well as increasing Th17 and moDDC infiltration in the skin. In addition, we performed oleic acid and stearic acid stimulation experiments on DCs in vitro to explore their regulation on DCs, and we found that stimulating with oleic acid and stearic acid increased the secretion of IL-23 in moDDCs. At last, K14-VEGF mice were treated with PDE-4 inhibitor to investigate changes in the intestinal microbiota following the alleviation of psoriasis-like skin phenotype and our results showed that PDE-4 inhibitor alleviated psoriasis-like phenotype accompanied by the recovery of intestinal microbiota, including increased *Parabacteroides distasonis* and decreased *Prevotella* in the colon of K14-VEGF mice.

It has been reported that, compared with healthy patients, the abundance of *Prevotella* in the intestines of patients with psoriasis is increased, while that of the *Porphyromonadaceae* family, to which *Parabacteroides distasonis* belongs, is decreased.^43,44^ In addition, a decrease in the abundance of *Parabacteroides* was observed in the gut of psoriatic patients, which is consistent with our findings.^11,45,46^

The occurrence of the disease is accompanied by disorders of intestinal microbiota. As we concluded in our previous review: *Akkermansia muciniphila*, a widely reported intestinal microbe that improves host metabolism, was decreased significantly in diabetic model mice and diabetic patients. Metformin treatment reversed type 2 diabetes-associated gut microbiota changes, with the composition of the gut microbiota, including *Akkermansia* levels, resembling nondiabetic controls.^47^ In addition, Tuhuaiyin, a kind of Chinese herb, was reported to alleviate IMQ-induced psoriasis-like phenotype companied with the remodels of the gut microbiota.^48^ In our study, we found that treatment with PDE-4 inhibitors contributed to the recovery of intestinal microbiota in psoriasis-like mice, including *Parabacteroides distasonis* and *Prevotella* levels. Administration of vancomycin depleted almost all gram-positive intestinal microorganisms, which led to an increased abundance of *Akkermansia muciniphila* and thus reduced the incidence of diabetes in NOD mice.^49^ In addition to antibiotics, defensin-related cryptdin-25(a synthesized peptide) has been reported to reduce the colonization of *Candidatus Arthromitus* and thus reduce Th17 response.^50^ In our finding, administration of gentamicin depleted the *Prevotella*, which led to an increased abundance *Parabacteroides distasonis* and thus improved psoriasis-like symptoms in mice which suggest that targeted depletion of the intestinal microbe has a great prospect for the treatment of psoriasis.

The intestinal mucus barrier is an important barrier for host resistance to external infection, and it is the main place where intestinal microorganisms co-exist with host cells. IL-17 has been reported to stimulate the trachea to secrete more mucus through the IL-6 paracrine/autocrine loop.^51^ *Prevotella* colonizes the mucus layer of mammals and can use mucus as a source of nitrogen and carbon. Thus, increased mucus production may explain the higher abundance of *Prevotella* in the intestines of mice with severe psoriasis-like skin phenotype.

*Parabacteroides* was reported to protect against the progression of seizures by modulating amino acid metabolism in the intestines and brain.^52^ Moreover, *Parabacteroides distasonis* was shown to reduce abnormal lipid metabolism and obesity by increasing the production of intestinal cholic acid.^53^ Thus, our results suggest that the reduction in the abundance of *Parabacteroides* leads to disturbances in intestinal fatty acid metabolism, which results in increased levels of certain intestinal fatty acids, such as oleic, stearic, and linoleic acids, thereby exacerbating psoriasis-like symptoms. Traumatic acid, a medium and long-chain fatty acid secreted by plants when damaged, has been reported to repair skin damage.^54,55^ Thus, the reduction in the levels of microbiota-derived traumatic acid that we have observed may also play an important role in psoriasis development.

Psoriasis, obesity, and other metabolic abnormalities may be closely related. A clinical study involving 35,000 participants showed that metabolic syndrome is associated with an increased risk of psoriasis.^56^ An analysis of the metabolic factors showed that obesity was the central factor in this association. Moreover, epidemiological studies have provided strong evidence suggesting that obesity and weight gain are risk factors for psoriasis.^57^ Nakamizo and colleagues found that the accumulation of IL-17A-producing γδT cells in psoriatic lesions of high-fat-diet-induced obese mice leads to the aggravation of psoriatic dermatitis. Palmitic acid and oleic acid can sensitize DCs, thus enhancing Th17 cell responses and promoting the secretion of IL-17, IL-22, and other proinflammatory cytokines. Furthermore, mice fed a high-fat diet containing oleic acid, stearic acid, and linoleic acid showed worsened IMQ-induced, Th1/Th17‐driven psoriatic dermatitis. Furthermore, serum total free fatty acids such as palmitic acid and oleic acid were closely related to the severity of skin inflammation.

Psoriasis is an autoimmune disease that seriously endangers human health for which there is no affordable and effective treatment.^58,59^ To develop better treatments, the pathogenesis of psoriasis must be urgently explored. As the second genome of the human body, the intestinal microbiome, which is composed of many species, has great potential for exploitation.^60^ *Lactococcus lactis* synthesizes vitamin K_2_, which cannot be synthesized by the human body, and is therefore transplanted to patients with vitamin K deficiency.^61^ In addition, oral administration of *Akkermansia muciniphila* has been shown to decrease body weight and the levels of blood markers related to liver dysfunction in overweight and obese humans.^62^ Thus, the administration of intestinal microorganisms has valuable clinical applications for the treatment of several diseases, including psoriasis. Administration of *Bifidobacterium adolescentis* CCFM667, *Bifidobacterium breve* CCFM1078, *Lacticaseibacillus paracasei* CCFM1074, and *Limosilactobacillus reuteri* CCFM1132 ameliorated psoriasis-like pathological characteristics in IMQ-induced psoriasis-like mice.^63^ In addition, results of clinical trials showed that *Bifidobacterium infantis* 35624 reduced systemic proinflammatory biomarkers in patients with psoriasis.^64^ As a new treatment, interventions of intestinal microbiology for psoriasis is promising. However, many interventions, including the administration of *Bifidobacterium* in the treatment of psoriasis, have unclear mechanisms. Therefore, more studies should be carried out to find intestinal microorganisms with potential therapeutic or aggravating effects on psoriasis, and further explore their interaction mechanism with psoriasis.

In conclusion, our study investigated the relationship between the intestinal microbial composition and pathogenesis of psoriasis-like phenotype in mice, and through metabolomics, demonstrated the mechanism whereby intestinal microorganisms may affect the development of psoriasis. Our results indicate that altered intestinal microbial composition and abnormal fatty acid metabolism may play a key role in the pathogenesis of psoriasiform skin inflammation, thus suggesting a new target for the clinical diagnosis and treatment of psoriasis.

## Materials and methods

### Ethics approval and consent to participate

The animal protocols were approved by the Committee on the Ethics of Animal Experiments of Sichuan University. The experimental procedures were conducted according to the ethical guidelines for the care and use of laboratory animals of the National Institutes of Health (https://grants.nih.gov/grants/olaw/guide-for-the-care-and-use-of-laboratory-animals.pdf) and the International Association for the Study of Pain (IASP). Every effort was made to decrease the number of animals used and to reduce animal suffering.

### Mice

K14-VEGF mice (FVB-Tg [Krt14-Vegfa]3Dtm/J) were purchased from Jackson Laboratories (Catalog no 005705). C57BL/6 mice (6 weeks old) and FVB/J mice (8 weeks old) were purchased from Vital River Laboratory Animal Technology Co., Ltd. The mice were housed under the following controlled conditions: a steady temperature of 25 ± 1 °C, a 12 h light/12 h dark cycle, and free access to food and water. Littermates of the same sex were randomly assigned to experimental groups.

### IMQ psoriasis-like skin model

C57BL/6 mice were shaved on the back 1 day before induction, and then 62.5 mg of Aldara cream (Sichuan MingXin Pharmaceutical Co., LTD., H20030129) containing 5% IMQ was evenly spread on the back daily for five days.

### PASI score

An objective scoring system was developed based on the clinical PASI, except that for the mouse model, the affected skin area is not taken into account in the overall score.^65^ Erythema, scaling, and thickening were scored independently on a scale from 0 to 4: 0, none; 1, slight; 2, moderate; 3, marked; 4, very marked. The level of erythema was scored using a scoring table with red taints. The cumulative score, including erythema (0–4), scaling (0–4), and thickening (0–4) served as a measure of the severity of inflammation (total scale 0–12). Mice were scored by two scorers who were blind to the experimental groups, and average scores of PASI were calculated.

### H&E staining

Ear and colon tissue were fixed in 4% paraformaldehyde in PBS, embedded in paraffin, sectioned, and stained with H&E for histopathologic examination. Images were captured using an Olympus BX600 microscope (Olympus Corporation, Tokyo, Japan) and a SPOT Flex camera (Olympus Corporation, Tokyo, Japan), and were analyzed with ImagePro Plus (version 6.0, Media Cybernetics) software. Epithelial thickness and cell infiltration were evaluated in independent regions. A pathological score based on the Baker score system was obtained to assess the severity of psoriasiform skin inflammation in ear tissues.

### RT-qPCR analysis

Total RNA from mice tissues was extracted with TRIzol agent (Thermo Fisher Scientific, 15596018) according to the manufacturer’s protocol. Gel electrophoresis was performed to examine the integrity of the total RNA extracted. After the genomic DNA elimination reaction, the total RNA (2 µg) was reverse transcribed into cDNA. A primeScript RT reagent kit with gDNA Eraser (Takara Bio, RR047A) was used for reverse transcription to produce cDNA at 37 °C for 15 min and at 85 °C for 5 s according to the manufacturer’s protocol. The obtained cDNA (20 ng) was subjected to RT-qPCR analysis with TB Green™ Premix Ex Taq™ II (Takara Bio, RR820) according to the manufacturer’s protocol. The results were normalized to beta-actin, and quantification was performed using the 2^−^^ΔΔCt^ method. The melting curves ensured the amplification of a single product. All primers were obtained from Chengdu Qing Ke Zi Xi Biotechnology Co. DNA of fecal samples was extracted using a Bacterial DNA Kit (TIANGEN, DP302). Sequences for qRT-PCR primers are listed in Supplemental Table 1.

### Western blotting

The samples derived from ears or skin were lysed, separated using SDS–PAGE gels (Beyotime Institute of Biotechnology), and transferred to polyvinylidene fluoride (PVDF) membranes (Merck Millipore). The proteins were incubated overnight with primary antibodies: mouse IL-17A (Abcam, ab79056, 1:1,000 dilution) and β-actin (CST, 4970, 1:1,000 dilution). Then, primary antibodies were labeled using a goat anti-rabbit antibody conjugated to horseradish peroxidase (HRP) (Invitrogen, A27036) and further detected using ECL reagents (Merck Millipore, WBKLS0500). The intensity of the band was quantified using ImageJ (National Institutes of Health).

### 16S rRNA gene microbiota profiling

Fecal samples were collected and stored at −80 °C. Samples were prepared and analyzed by Beijing Genomics Institute, Inc, and Shanghai Bioprofile Biological Technology Co., LTD. Bacterial genomic DNA was extracted from mouse fecal samples using PowerSoil Kit (MoBio, 12888–50). The V4 regions of the 16S rRNA gene were PCR amplified using individually barcoded universal primers and 30 ng of the extracted genomic DNA. The PCR reaction was set up in triplicate, and the PCR product was purified using Agencourt AMPure XP beads. The purified PCR product was pooled in equal molar concentrations, quantified by the Agilent 2100 Bioanalyzer, and sequenced using the HiSeq Illumina 2500 platform. OTUs were chosen by open-reference OTU picking based on 97% sequence similarity to the Greengenes 13_5 database. Taxonomy assignment and rarefaction were performed using QIIME1.8.0. for data analysis, including PcoA and hierarchical clustering was performed using R.

### AB-PAS staining

Colon tissues were fixed in 4% paraformaldehyde in PBS, embedded in paraffin, sectioned, and stained with AB-PAS (Solarbio, G1285). Images of the sections were captured to count the number of goblet cells per crypt.

### Co-housing

Three 2 M K14-VEGF mice were co-housed with three 6 M K14-VEGF mice per cage. After 4 weeks of co-housing, mice were sacrificed to harvest the ear and colon tissue. Fecal samples were collected at the end of the co-housing period and stored at −80 °C for 16S rRNA gene sequencing.

### Immunohistochemistry

Colon tissues were fixed in 4% paraformaldehyde in PBS, and the fixed sections were incubated in 3% H_2_O_2_ solution in PBS at room temperature for 10 min. Antigen retrieval was performed in sodium citrate buffer (0.01 M, pH 6.0) in a microwave oven at 1000 W for 3 min. Nonspecific antibody binding was blocked by incubation with 5% normal goat serum in PBS for 1 h at 25 °C. Slides were stained overnight at 4 °C with anti-claudin-3 antibody (Invitrogen, 34–1700, 1:200 dilution) and anti-CD45 antibody (BioLegend, 103102, 1:1000 dilution). The slides were subsequently washed and incubated with biotin-conjugated secondary antibodies for 30 min, and then with horseradish peroxidase streptavidin (HRP Streptavidin) for 30 min (SPlink Detection Kits; ZSGB-BIO, SP-9001 or SP-9002). The sections were developed using a 3,3’-Diaminobenzidine (DAB) substrate kit (ZSGB-BIO, ZLI-9017) and counterstained with hematoxylin. Images were captured using an Olympus BX600 microscope and a SPOT Flex camera. ImagePro Plus was used for further quantification of the DAB intensity in the image.

### Fecal metabolomics

Mice were fasted for 12 h and then fed for 4 h. After that, fecal samples were collected, frozen in liquid nitrogen immediately, and stored at −80 °C. Samples were prepared and analyzed in LC/MS platforms by Beijing Genomics Institute, Inc. In short, 100 µl samples were extracted by directly adding 300 µl of precooled methanol and acetonitrile (2:1, v/v), to which internal standards mix 1 (IS1) and internal standards mix 2 (IS2) were added for quality control of the sample preparation. After vortexing for 1 min and incubating at −20 °C for 2 h, the samples were centrifuged for 20 min at 3000 × *g*, and the supernatant was then transferred for vacuum freeze drying. The metabolites were resuspended in 150 µl of 50% methanol and centrifuged for 30 min at 4000 rpm, and the supernatants were transferred to autosampler vials for LC-MS analysis. A quality control (QC) sample was prepared by pooling the same volume of each sample to evaluate the reproducibility of the whole LC-MS analysis. The samples were analyzed on a Waters 2D UPLC (Waters, USA), coupled to a Q-Exactive mass spectrometer (Thermo Fisher Scientific, USA) with a heated electrospray ionization (HESI) source and controlled by the Xcalibur 2.3 software program (Thermo Fisher Scientific, Waltham, MA, USA). Chromatographic separation was performed on a Waters ACQUITY UPLC BEH C18 column (1.7 μm, 2.1 mm × 100 mm, Waters, USA), and the column temperature was maintained at 45 °C. The mobile phase consisted of 0.1% formic acid (A) and acetonitrile (B) in the positive mode, and 10 mM ammonium formate (A) and acetonitrile (B) in the negative mode. The gradient conditions were as follows: 0–1 min, 2% B; 1–9 min, 2–98% B; 9–12 min, 98% B; 12–12.1 min, 98% B to 2% B; and 12.1–15 min, 2% B. The flow rate was 0.35 ml/min and the injection volume was 5 μl.

The mass spectrometric settings for positive/negative ionization modes were as follows: spray voltage, 3.8–3.2 kV; sheath gas flow rate, 40 arbitrary units (arb); aux gas flow rate, 10 arbs; aux gas heater temperature, 350 °C; capillary temperature, 320 °C. The full scan range was 70–1050 m/z with a resolution of 70,000, and the automatic gain control (AGC) target for MS acquisition was set to 3e6 with a maximum ion injection time of 100 ms. The top three precursors were selected for subsequent MSMS fragmentation with a maximum ion injection time of 50 ms and resolution of 17,500, and the AGC was 1e5. The stepped normalized collision energy was set to 20, 40, and 60 eV. Data analysis, including principal component analysis and hierarchical clustering was performed using R.

### Cocktail antibiotic treatment

Before FMT, mice were administered with antibiotics to reduce the intestinal microbiota. Mice were treated for 5 consecutive days with 200 μl of an antibiotic cocktail (with each daily dose being administered by oral gavage after a 6 h fast) containing 1 g/L ampicillin, 0.5 g/L neomycin, 0.5 g/L vancomycin, and 1 g/L metronidazole. Fecal samples were collected before and after antibiotic treatment to verify the effect.

### Gentamicin treatment

Mice were administered with gentamicin to reduce the abundance of *Prevotella*. Mice were gavaged with 200 μl of gentamicin (1 g/L, twice a day) for 15 days.

### Bacterial FISH

Colon tissues were fixed in 4% paraformaldehyde in PBS, embedded in paraffin, and sectioned. Sections were pretreated with the FISH probe reaction buffer kit (Genepharma, F26501/100) and then hybridized with the probes. The probes were Prv392:^19^ 5′ Texas Red-GCACGCTACTTGGCTGG and PD:^20^ 5′ FAM- CAGCGATGAATCTTTAGCAAATATCC. Sections were incubated in 2 μM FISH probes (Qing Ke Zi Xi Biotechnology Co) for 14 h at 37 °C in a humidified chamber. After that, sections were then incubated in the dark with DAPI for 10 min at room temperature. Images were analyzed using a Leica DM RXA2 confocal microscope controlled by Leica Microsystems confocal software (version 2.61 Build 1537; Leica Microsystems, Wetzlar, Germany). Sequences for probes are listed in Supplemental Table 1.

### Cell preparation

Mouse BM-derived DCs were isolated from C57BL/6 mice. The bone marrow was rinsed into the medium (RPMI 1640, Invitrogen, 31870082), filtered through a 70 μm sieve, and then centrifuged. Cell density was adjusted to 1 × 10^6^/ml and cultured in DC medium (RPMI 1640, Invitrogen, 31870082) supplemented with 5% fetal bovine serum (Invitrogen,10100147), 1% penicillin/streptomycin, 1% l-glutamine amide (Invitrogen, 10378016), 10 ng/ml recombinant GM-CSF (Sino Biological, 51048-MNAH), 20 ng/ml recombinant IL-4 (Sino Biological, 51084-MNAE) for 7 days at 37 °C in an incubator containing 5% CO_2_. After culture, flow cytometry was used to identify the induced differentiation results and follow-up experiments were carried out. After that, a total of 2 × 10^5^ mouse DCs were stimulated with 200–800 μM BSA-OA or BSA-SA complexes for 6 h, then when indicated, they were activated in the presence of 100 ng/ml LPS (Sigma-Aldrich, L2630).^38^

### ELISA

Cell culture supernatants were collected and mouse IL-23(p19) A were detected by ELISA using Mouse Interleukin 23 (IL-23) Kit (Shuangyingbio, SY-M06009).

### Fecal microbiota transplantation

A fecal slurry was obtained by pooling fecal pellets from six to eight donor mice (two fresh feces pellets per mice). Feces pellets were resuspended with a vortex in 600 μl of reduced PBS (PBS with 0.5 g/L cysteine and 0.2 g /L Na_2_S) and then filtered through a 70 μm cell strainer to remove insolubilized material. About 100 μl of supernatant was administered to each mouse by oral gavage three times a week for 2 weeks. After this 2-week period, mice received the microbiota suspension once a week until natural death or sacrifice.

### Preparation of single-cell suspensions

Single-cell suspension preparation and flow cytometry were based on our previously published methods.^66^ Ear tissues were incubated for approximately 1.5 h at 37 °C with 5 ml of RPMI medium (Thermo Fisher Scientific, C22400500BT) containing 1 mg/ml of Collagenase type IV (Gibco, 17100-017). Then, the tissues were minced with sharp scissors and incubated for an additional 15 min with 0.1 mg/ml DNase (Roche, 10104159001). Single-cell suspensions were made by mechanical dissociation with a gentleMACS dissociator (Miltenyi Biotech, Bergisch Gladbach, Germany) and filtered sequentially through 40 and 70 μm cell strainers (BD Bioscience, 352340 and 352350). Cells were washed once with PBS.

For DLNs, single-cell suspensions were harvested by pressing the tissues through a 70 µm mesh in 5 ml PBS.

For colonic lamina propria, the colonic contents were first rinsed with precooled PBS. Then, 0.3 ml of digestive solution 1 (1640 RPMI medium containing 5 mM EDTA, 1 mM DTT, and 5% FBS) was added into a 1.5 ml EP tube, and the colon tissue was cut into pieces. Then, the shredded colon tissue was transferred to a 15 ml centrifuge tube containing 5 ml of digestive solution 1 and shaken and incubated at 37 °C for 30 min. After mixing, the suspension was left to stand for 1 min, the supernatant was discarded, and 6 ml of digestive solution 2 (1640 RPMI medium containing 0.2% type IV collagenase, 0.025% nuclease, and 5% FBS) was added. The mixture was shaken and incubated at 37 °C for 40–60 min, during which the liquid pipette was gently blown every 20 min. Finally, the single-cell suspension was filtered through a 70 μm filter.

### Flow cytometry

For surface staining, cells were stained with appropriate antibodies against surface antigens diluted in PBS on ice for 30 min. Cellular viability was assessed by staining with 7-aminoactinomycin D (7-AAD) (BioLegend, 420404) to exclude dead cells. For the analysis of cytokines production, in vitro re-stimulation and intracellular staining, single-cell suspensions were incubated for 4 h at 37 °C with PMA (Sigma-Aldrich, p1585; 200 ng/ml), brefeldin A (BioLegend, 420601; 5 µg/ml), and ionomycin (Abcam, ab120116; 1 µg/ml). The cells were then washed and stained with the fixable viability stain 620 (FVS 620-PE-Texas Red; BD-Biosciences, 564996) for 10 min. After performing surface staining as described above, cells were fixed with 4% paraformaldehyde and permeabilized with PBS supplemented with 0.1% Triton X-100. Intracellular staining with fluorescent-labeled antibodies was performed for 30 min in PBS. For transcription factor staining, cells were treated using a transcription factor buffer set (BD, 562574), and then staining with fluorescent-labeled antibodies was performed for 30 min. For flow cytometric analysis, the cells were washed and resuspended in PBS. Flow cytometry was performed using a NovoCyte flow cytometer controlled by ACEA NovoExpress™ software (ACEA Biosciences, San Diego, CA, USA), and a BD LSRFortessa flow cytometer controlled by FlowJo TM 10 software (BD, 647800L6). The single-cell suspensions were stained with the following antibodies: CD3-APC-CY7 (BioLegend, 100222), IL-17A-PE (BioLegend, 512305), CD4-PerCP-Cy5.5 (BioLegend, 100434), CD8-BV786 (BioLegend, 100750), CD45-BV510 (BioLegend, 103138), CD11b-APC (BioLegend, 101212), MERTK-PE (BioLegend, 151506), MHCII-FITC (BioLegend, 107606), CD64-PE-CY7 (BioLegend, 139314), Ly6c-APC-Cy7 (BioLegend, 128026); CD4-PE (BioLegend, 100408), CD3- FITC (BD, 100204), IL-17A-PerCP (BioLegend, 506920), CD8-APC-CY7 (BioLegend, 344714), IFN-γ-FITC (BioLegend, 505806), Foxp3-Alexa Fluor-488 (Biolegend,320011), IL-4-PE-Cy7 (BioLegend, 504117). Antibodies were used at 1:100 dilution.

### Administration of apremilast

4–5 M mice (18 weeks) were gavaged with 200 μl of apremilast (Selleck Chemicals, CC-10004) preparation (20 ml PEG400, 50 mg apremilast, 0.5 ml tween-80, 79.5 ml H_2_O) or vehicle preparation (20 ml PEG400, 0.5 ml tween-80, 79.5 ml H_2_O) daily following 5 days per week for 6 weeks.

### Supplementation with oleic acid and stearic acid

Mice were gavaged with oleic acid (50 mg/ml) and stearic acid (20 mg/ml) in PBS according to body weight (10 μl/g).^67,68^ After supplementation with oleic acid and stearic acid for 41 days, mice were sacrificed to harvest the ears, skin, and DLNs.

### Statistical analysis

Statistical details are provided in the figure legends. All statistical analysis was performed with GraphPad Prism 8 software (GraphPad Software Company, version 8.0.0). Student’s *t*-test and one-way ANOVA was used for comparing the groups. *P* < 0.05 was considered statistically significant.

## Supplementary information

Supplementary Materials

## Data Availability

The data that support the findings of this study are openly available in [NCBI-SRA] at [https://dataview.ncbi.nlm.nih.gov/object/PRJNA771247?reviewer=40enahaki9tr9t1qs1aacpp7m9] and reference number [PRJNA771247, 770517, and 804695.].
