# Supplementary material for: Intestinal dysbiosis exacerbates the pathogenesis of psoriasis-like phenotype through changes in fatty acid metabolism
Source: Signal Transduct Target Ther. 2023 Jan 30;8:40. doi: 10.1038/s41392-022-01219-0 (PMC9884668; doi:10.1038/s41392-022-01219-0)
Supplement: Supplementary file 1 — Supplementary Materials [file 41392_2022_1219_MOESM1_ESM.docx]

Supplementary Materials for

Intestinal dysbiosis exacerbates the pathogenesis of psoriasis-like phenotype through changes in fatty acid metabolism

Qixiang Zhao,^a#^ Jiadong Yu,^a#^ Hong Zhou,^a#^ Xiaoyan Wang,^a#^ Chen Zhang,^a^ Jing Hu,^a^ Yawen Hu,^a^ Huaping Zheng,^a^ Fanlian Zeng,^a^ Chengcheng Yue,^a^ Linna Gu,^a^ Zhen Wang,^a^ Fulei Zhao,^a^ Pei Zhou,^a^ Haozhou Zhang,^a^ Nongyu Huang,^a^ Wenling Wu,^a^ Yifan Zhou,^a^ and Jiong Li^a*^

Correspondence to: Jiong Li (lijionghh@scu.edu.cn)

**This PDF file includes:**

Figures. S1 to S9

Table. S1


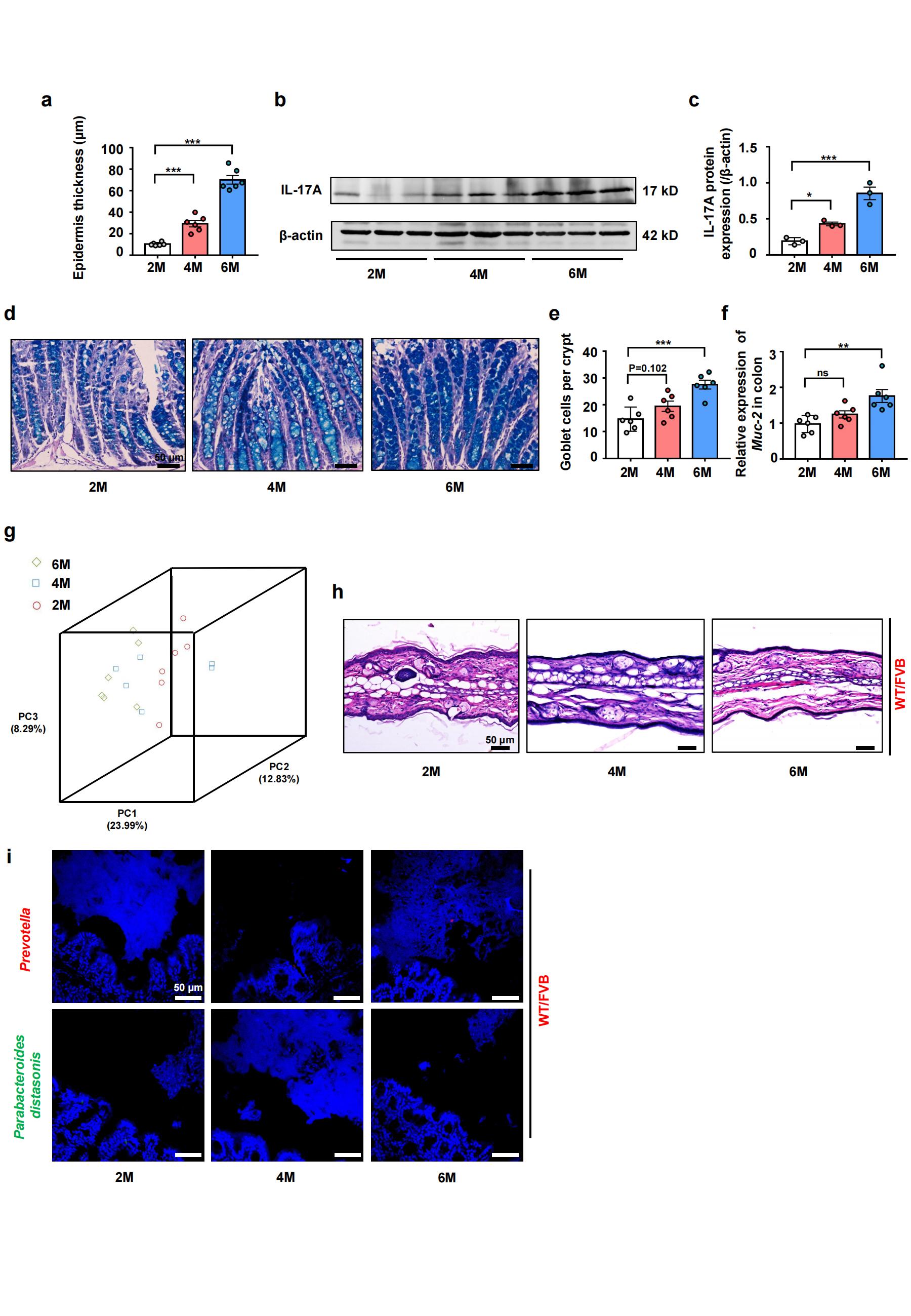


Figure. S1.

**Severity of psoriasis-like skin phenotype increases with age in K14-VEGF transgenic mice, and this is accompanied with changes in the composition of the intestinal microbiota. a**. Average epidermal thickness of 2M, 4M, and 6M mice. **b**. Representative western blot bands indicating IL-17A in the ears of 2M, 4M, and 6M mice. **c**. Relative expression of IL-17A protein in the ears of 2M, 4M and 6M mice. **d**. Representative AB-PAS staining of colons of 2M, 4M, and 6M mice. (Scale bars: 50 μm). **e**. Quantitation of average goblet cells per crypt. **f**. Relative mRNA expression of *Muc-2* in the colon of 2M, 4M, and 6M mice. **g**. Principal coordinates analysis (PcoA) of unweighted UniFrac distance based on 16s rDNA profiling of feces from 2M, 4M, and 6M mice. **h**. Macroscopic characteristics of the ears in 2M, 4M, and 6M mice (WT/FVB). **i**. Representative fluorescence in situ hybridization for *Prevotella* (Prv392) and *Parabacteroides distasonis* (PD) in the colon of 2M, 4M, and 6M mice (WT/FVB). Data presented as mean ± SD on relevant graphs. ∗*P* ≤ 0.05; ∗∗*P* ≤ 0.01; ∗∗∗*P* ≤ 0.005 (one-way ANOVA). (n=6) in (**a**) and (**d**)-(**g**). (n=3) in (**b**) and (c). (n=4) in (**h**) and (**i**); ns, not significant.


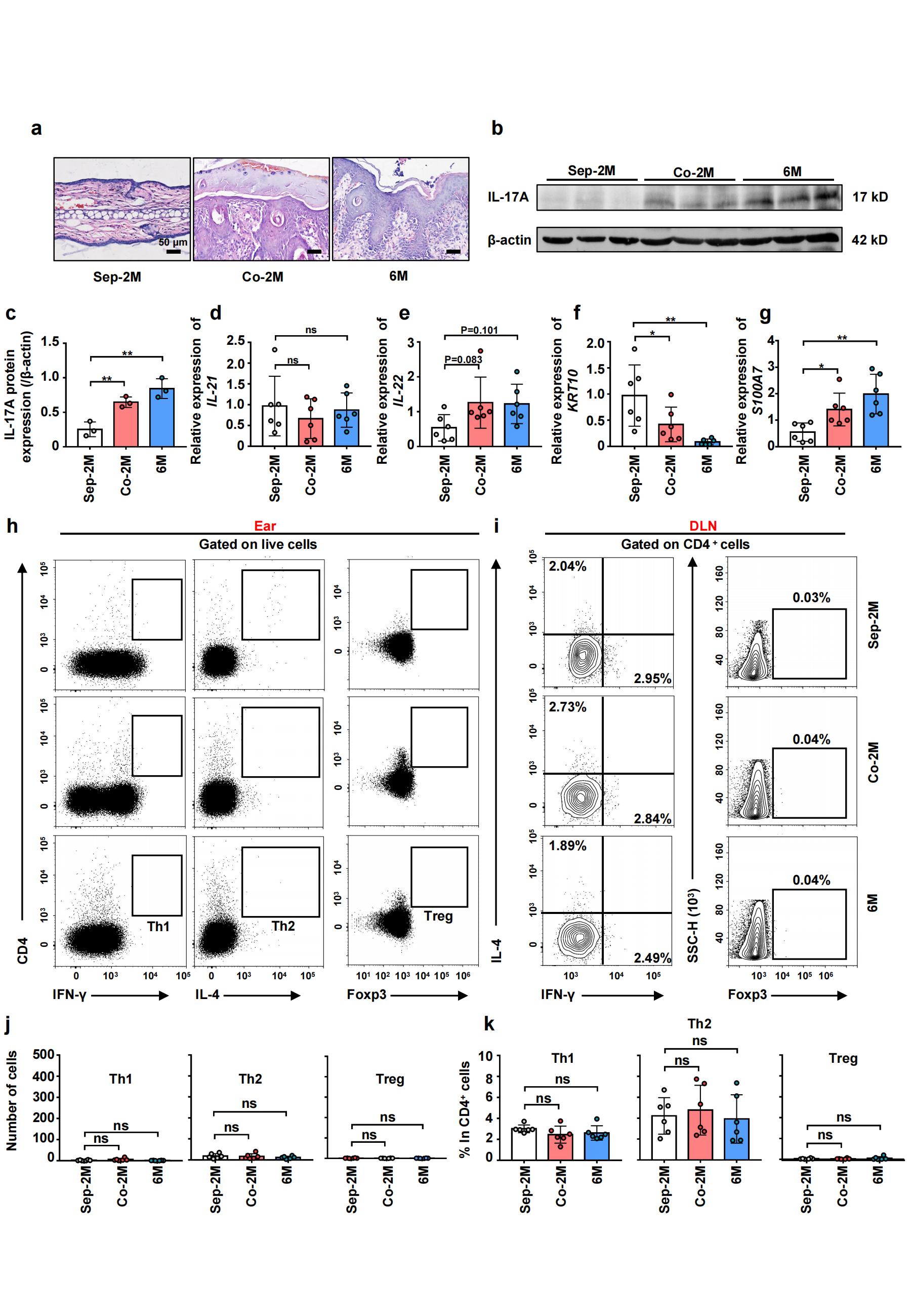
Figure. S2.

**Co-housing with 6M mice exacerbated psoriasis-like skin phenotype in, and changed the intestinal microbiota composition of, 2M mice**. **a**. Representative H&E staining of ears in Sep-2M, Co-2M, and 6M mice. (Scale bars: 50 μm). **b**. Representative western blot bands indicating IL-17A in the ears of Sep-2M, Co-2M, and 6M mice. **c**. Relative expression of IL-17A protein in the ears of Sep-2M, Co-2M, and 6M mice. **d-g**. Relative mRNA expression of *IL-21*, *IL-22*, *KRT10*, and *S100A7* in the ears of Sep-2M, Co-2M, and 6M mice. **h**. Analysis of Th1, Th2 and Treg cells by flow cytometry in ears. **i**. Analysis of Th1, Th2 and Treg cells by flow cytometry in DLNs. **j**. Number of Th1, Th2 and Treg cells in ears. **k**. Percentage of Th1, Th2 and Treg cells in CD4^+^ cells in DLNs. Data presented as mean ± SD on relevant graphs. ∗*P* ≤ 0.05; ∗∗*P* ≤ 0.01; ∗∗∗*P* ≤ 0.005 (one-way ANOVA). (n=6) in (**a**) and (**d**)-(**k**). (n=3) in (**b**)-(**c**); ns, not significant.


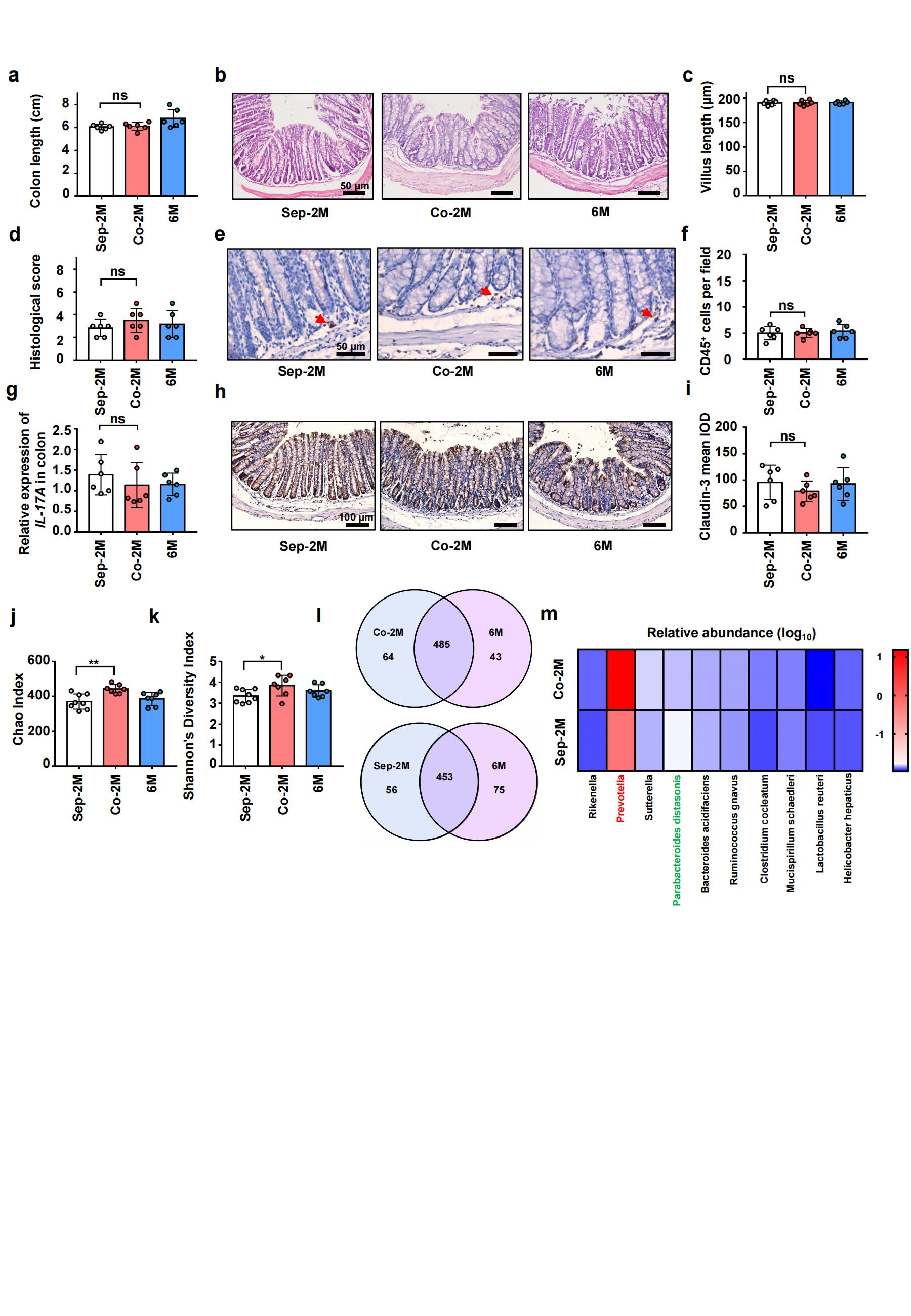
 Figure. S3.

**Co-housing with 6M mice changed the intestinal microbiota composition of 2M mice but not affecting the intestinal barrier and inflammation. a**. Colon length. **b**. Representative H&E staining of colons of Sep-2M, Co-2M, and 6M mice. (Scale bars: 50 μm). **c**. Villus length. **d**. Cumulative histological score of colons. **e**. Representative micrographs of colon sections staining of anti-CD45 antibody. **f**. Quantification of the number of CD45^+^ cells per field in the colon. **g**. Relative mRNA expression of *IL-17A* in the colons of Sep-2M Co-2M, and 6M mice. **h**. Representative micrographs of colon sections staining of anti-claudin-3 antibody. **i**. Median values of mean IOD of claudin-3 in Sep-2M Co-2M, and 6M mice. **j**. Total richness of intestinal microbiota was measured by Chao Index. **k**. Bacterial diversities were measured by the Shannon’s Index. **l**. Venn diagrams showing the numbers of shared and unique OTUs detected in Sep-2M Co-2M, and 6M mice. **m**. Taxonomic distributions of bacteria. Data presented as mean ± SD on relevant graphs. ∗*P* ≤ 0.05; ∗∗*P* ≤ 0.01; ∗∗∗*P* ≤ 0.005 (one-way ANOVA). (n=6) in (**a**)-(**i**). Sep-2M (n=8), Co-2M (n=7) and 6M (n=7) in (**j**)-(**m**); ns, not significant.


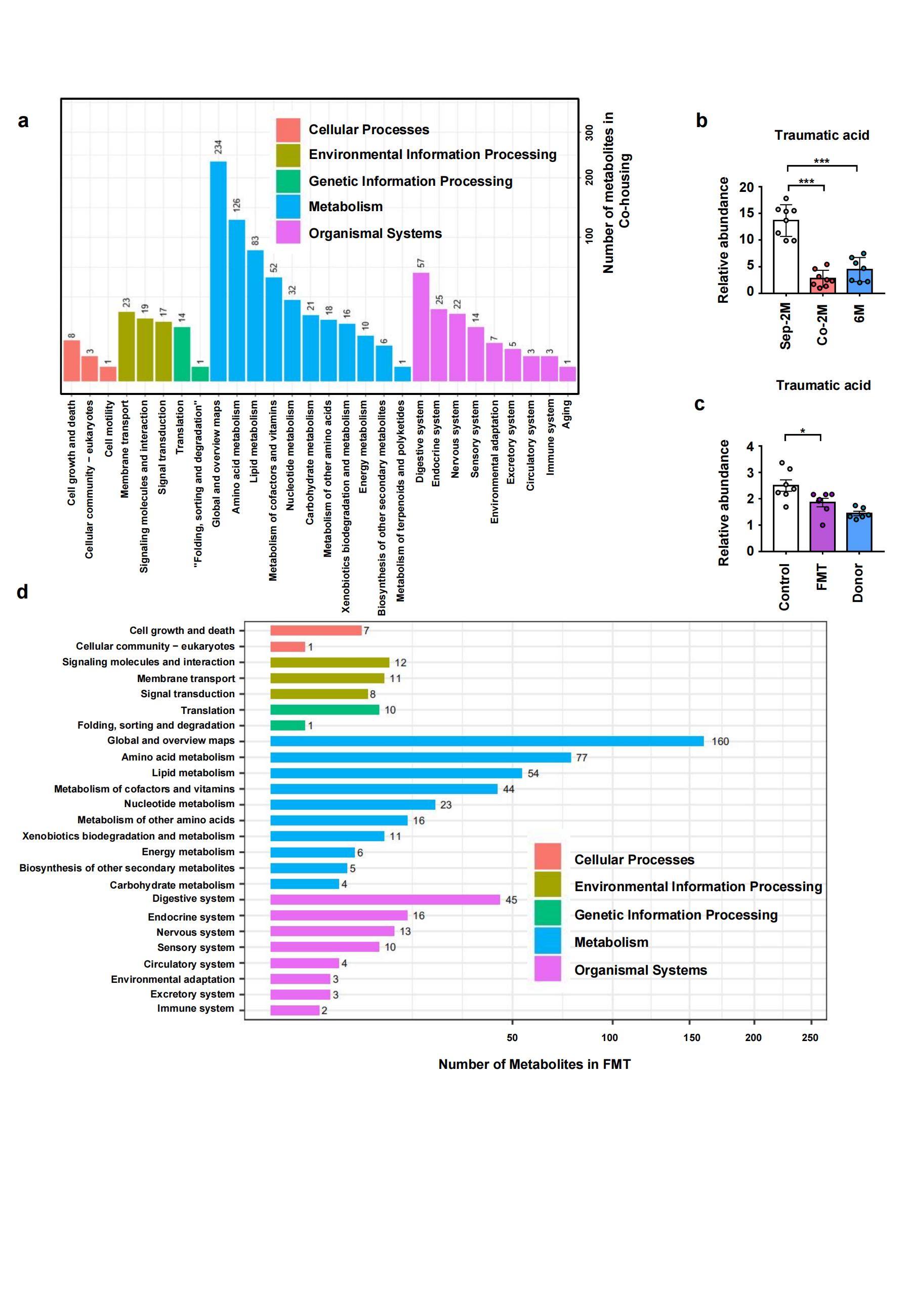


Figure. S4.

**Classification of fecal metabolites from co-housing and FMT experiment. a**. KEGG pathway classification chart of fecal metabolites from co-housing experiment. **b**. Relative abundance of Traumatic acid. **c**. Relative abundance of Traumatic acid. **d**. KEGG pathway classification chart of fecal metabolites from FMT experiment. Data presented as mean ± SD on relevant graphs. ∗*P* ≤ 0.05; ∗∗*P* ≤ 0.01; ∗∗∗ *P* ≤ 0.005 (one-way ANOVA). Sep-2M (n=8), Co-2M (n=8) and 6M (n=7). Control (n=7), FMT (n=7) and Donor (n=6).


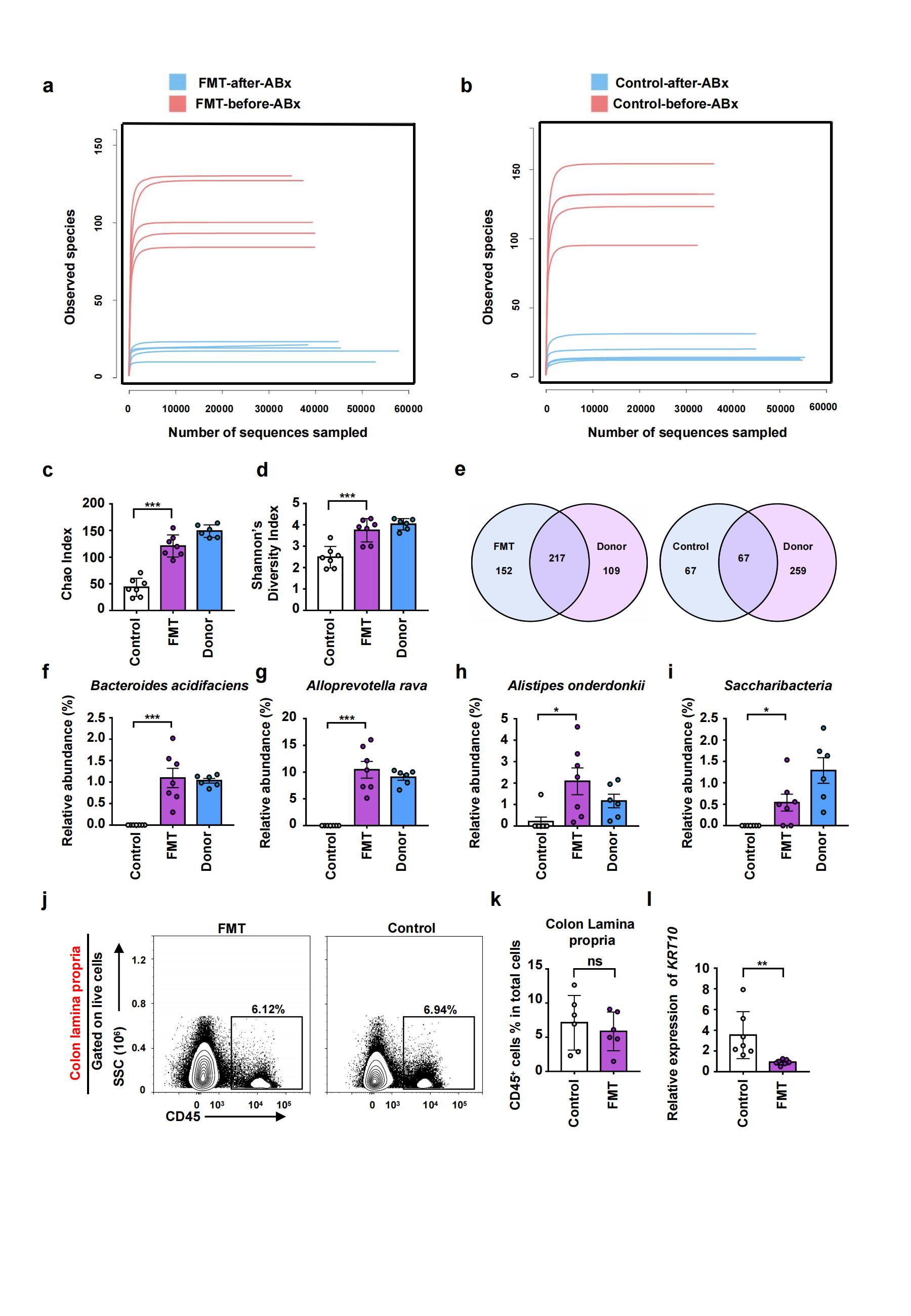


Figure. S5.

**FMT changed the composition of intestinal microbiota in 2M mice but not affecting the infiltration of colonic immune cells. a**. Observed species of FMT mice before and after antibiotic treatment. **b**. Observed species of Control mice before and after antibiotic treatment. **c**. Total richness of intestinal microbiota was measured by Chao Index. **d**. Bacterial diversities were measured by the Shannon’s Index. **e**. Venn diagrams showing the numbers of shared and unique OTUs detected in FMT, Control, and Donor mice. **f-i**. Fecal contents in FMT, Control, and Donor mice were analyzed for *Bacteroides acidifaciens*, *Alloprevotella rava*, *Alistipes onderdonkii* and *Saccharibacteria* colonization by 16S microbial sequencing. **j**. Analysis of immune cells (CD45^+^) by flow cytometry in colon lamina propria. **k**. Percentage of CD45^+^ cells in total cells. **l**. Relative mRNA expression of *KRT10* in ears. Data presented as mean ± SD on relevant graphs. ∗*P* ≤ 0.05; ∗∗*P* ≤ 0.01; ∗∗∗*P* ≤ 0.005. Two-tailed Student’s T-test was used in (**j**)-(**l**) and one-way ANOVA was used in (**c**)-(**i**). (n=5) in (**a)** and (**b)**. (n=6) in (**j**) and (**k**). Control (n=7), FMT (n=7) and Donor (n=6) in (**c**)-(**i**). (n=7) in (**l**); ns, not significant.


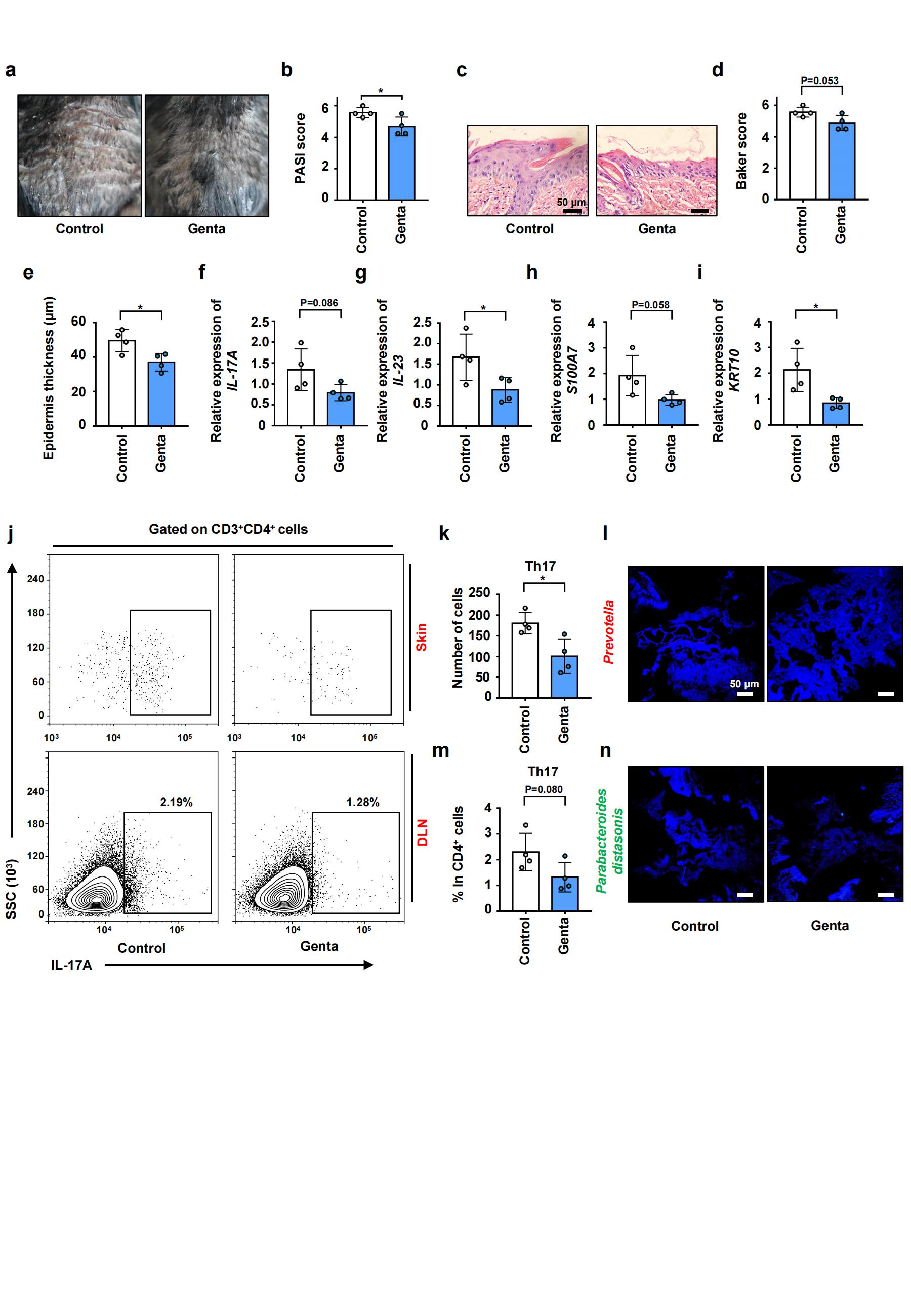
Figure. S6.

**Administration of gentamicin alleviated the pathogenesis of psoriasis-like skin phenotype in IMQ-induced psoriasis-like mice with the decreased infiltration of Th17**. **a**. Macroscopic characteristics of the skin in Control and Genta group mice. **b**. PASI score of skin in Control and Genta group mice. **c**. Representative H&E staining of skin. (Scale bars: 50 μm). **d**. Pathological score of skin sections using the Baker scoring system. **e**. Average epidermal thickness. **f-i** Relative mRNA expression of *IL-17A*, *IL-23*, *S100A7* and *KRT10* in the skin of Control and Genta group mice. **j**. Analysis of Th17 cells (CD4^+^ IL-17^+^) by flow cytometry in skin and DLNs. **k**. Number of Th17 cells in skin. **l**. Representative fluorescence in situ hybridization for *Prevotella* (Prv392) in colonic contents. **m**. Percentage of Th17 cells in CD4^+^ cells. **n**. Representative fluorescence in situ hybridization for *Parabacteroides distasonis* (PD) in colonic contents. Data presented as mean ± SD on relevant graphs. ∗*P* ≤ 0.05; ∗∗*P* ≤ 0.01; ∗∗∗*P* ≤ 0.005 (Two-tailed Student’s T-test). (n=4); ns, not significant.


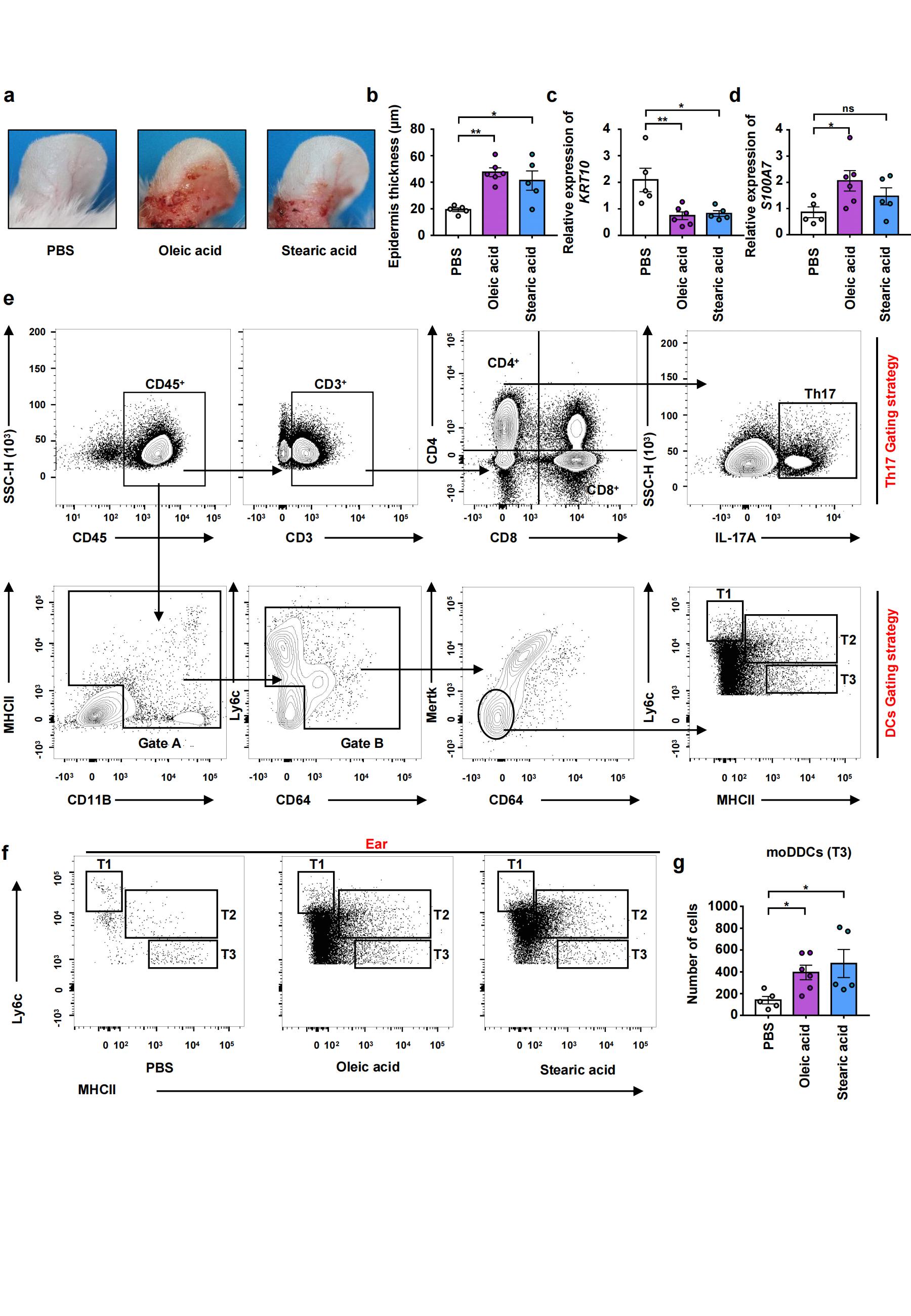


Figure. S7.

**Administration of free fatty acid exacerbated the pathogenesis of psoriasis-like skin phenotype of IMQ-induced psoriasis-like mice and promoted the differentiation of Th17 in DLNs as well as increased the infiltration of moDDCs and Th17 in skin.** **a**. Macroscopic characteristics of the ears in PBS, Oleic acid, and Stearic acid group mice. **b**. Average epidermal thickness. **c** and **d**. Relative expression levels of *KRT10* and *S100A7* in the ears of PBS, Oleic acid, and Stearic acid group mice. **e**. Flow cytometry plots, and gating strategy for the identification of monocytes (T1) and moDDCs (T2 and T3) in ear and Th17 in ear and DLN. **f**. Analysis of monocytes (T1) and moDDCs (T2 and T3) by flow cytometry in ears. **g**. Number of moDDCs (T3) in ears. Data presented as mean ± SD on relevant graphs. ∗*P* ≤ 0.05; ∗∗*P* ≤ 0.01; ∗∗∗*P* ≤ 0.005 (one-way ANOVA). PBS (n=5), Oleic acid (n=6) and Stearic acid (n=5).


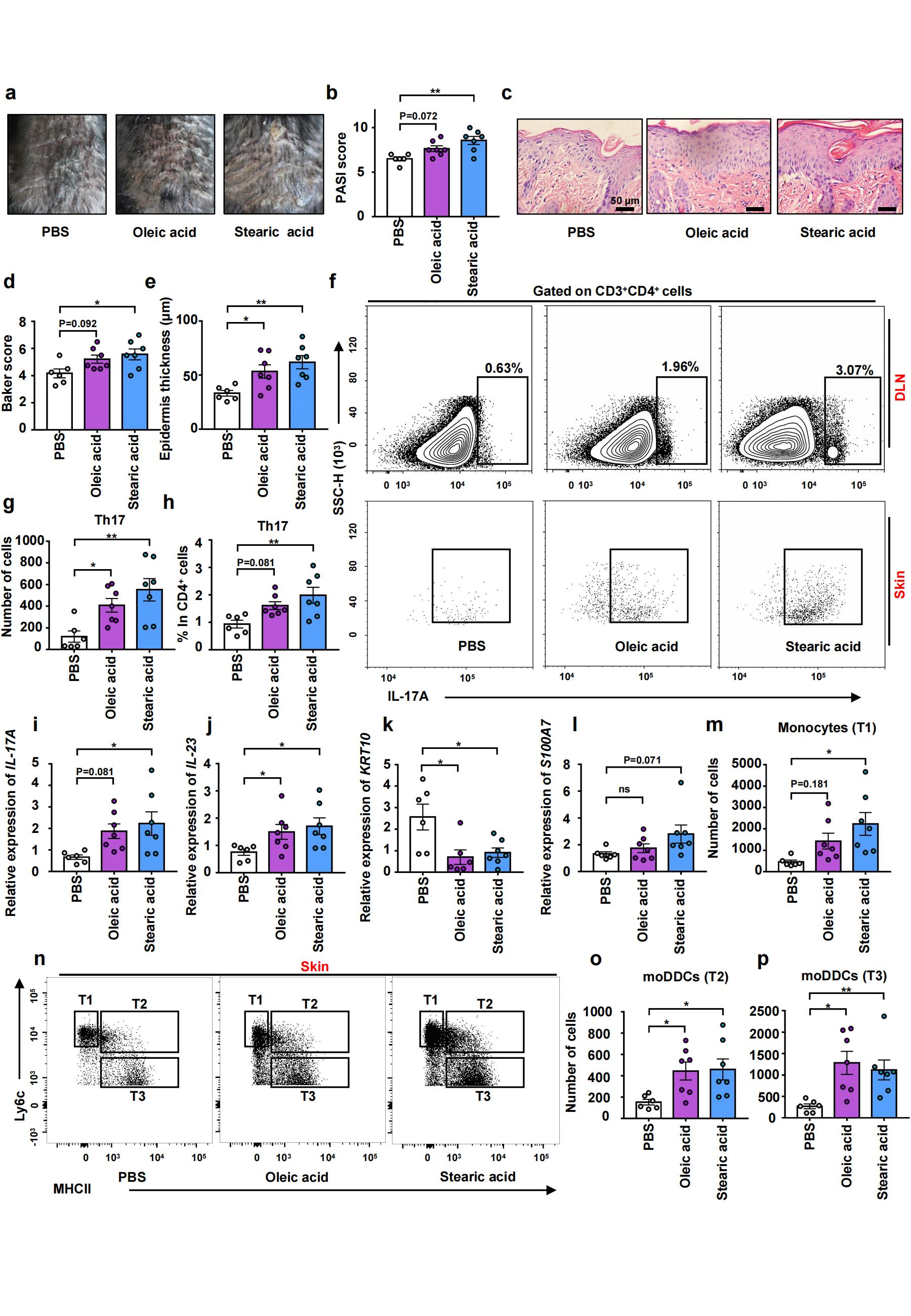
Figure. S8.

**Administration of free fatty acid exacerbated the pathogenesis of psoriasis-like skin phenotype of IMQ-induced psoriasis-like mice and promoted the differentiation of Th17 in DLNs as well as increased the infiltration of moDDCs and Th17 in skin.** **a**. Macroscopic characteristics of the skin in PBS, Oleic acid, and Stearic acid group mice. **b**. PASI score of skin. **c**. Representative H&E staining of skin (Scale bars: 50 μm). **d**. Pathological score of skin sections using the Baker scoring system. **e**. Average epidermal thickness. **f**. Analysis of Th17 cells (CD4^+^ IL-17^+^) by flow cytometry in DLNs and skin. **g**. Number of Th17 cells in skin. **h**. Percentage of Th17 cells in CD4^+^ cells in DLNs. **i-l**. Relative expression levels of *IL-17A*, *IL-23*, *S100A7* and *KRT10* in the skin of PBS, Oleic acid, and Stearic acid group mice. **m**. Number of Monocytes in skin. **n**. Analysis of monocytes (T1) and moDDCs (T2 and T3) by flow cytometry in skin. **o** and **p**. Number of moDDCs in skin. Data presented as mean ± SD on relevant graphs. ∗*P* ≤ 0.05; ∗∗*P* ≤ 0.01; ∗∗∗*P* ≤ 0.005 (one-way ANOVA). PBS (n=6), Oleic acid (n=7), and Stearic acid (n=7).


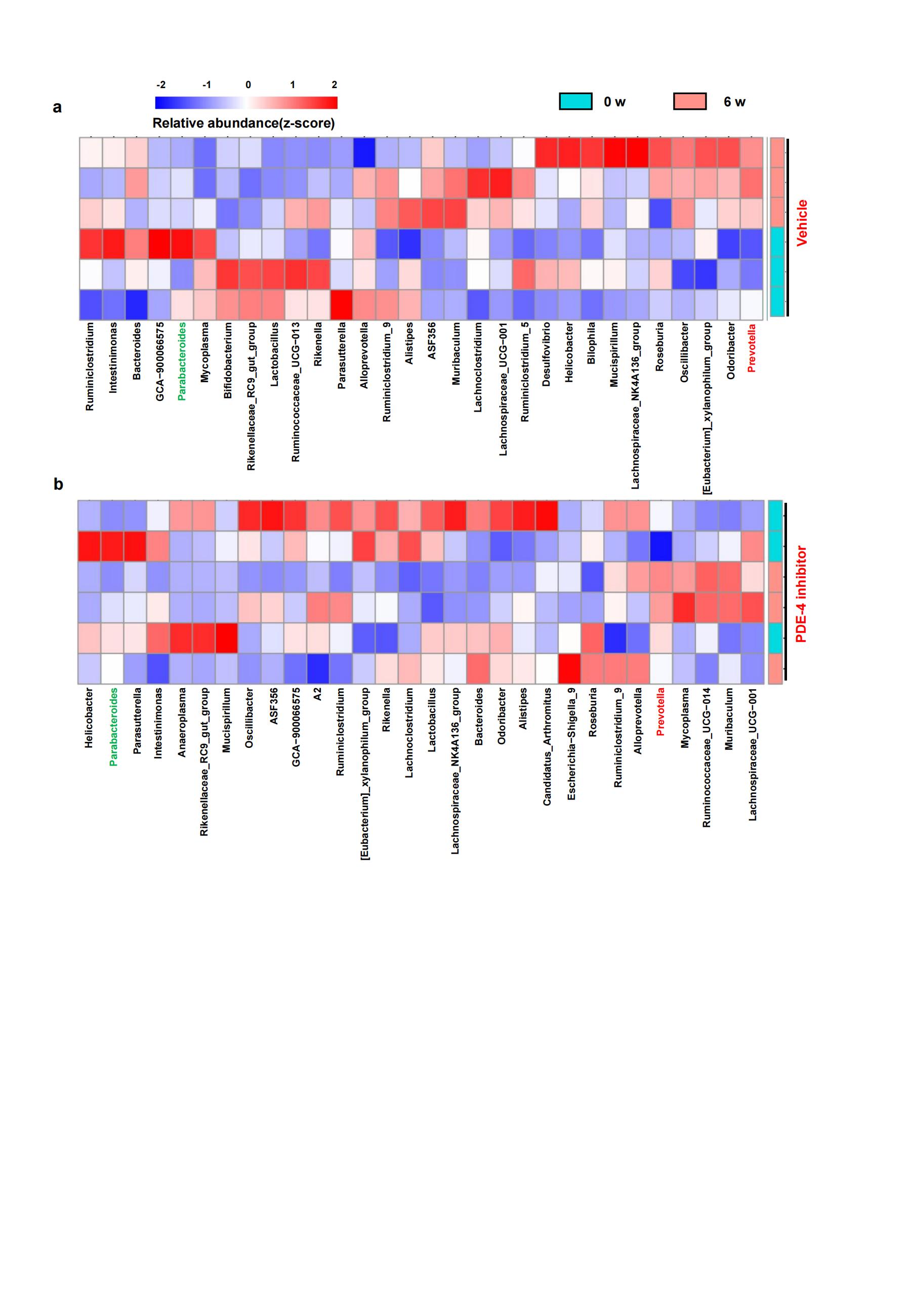
Figure. S9.

**Taxonomic distributions of bacteria in mice with the treatment of 0 week and 6 weeks.** **a**. Taxonomic distributions of bacteria in control mice. **b**. Taxonomic distributions of bacteria in PDE-4 treated mice. (n=3).

Table. S1.

**Real time qPCR primers and FISH probes sequences.**

| **Gene symbol** | **5' primer** | **3' primer** |
| --- | --- | --- |
| IL-17A | CTCAGACTACCTCAACCGTTCC | CATGTGGTGGTCCAGCTTTCC |
| IL-23p19 | CACCTCCCTACTAGGACTCAGC | TGGGCATCTGTTGGGTCT |
| IL-21 | GGAGACTCAGTTCTGGTGGC | GAGCGTCTATAGTGTCCGGC |
| IL-22 | TTTCCTGACCAAACTCAGCA | CTGGATGTTCTGGTCGTCAC |
| KRT10 | GGACCTTAAGGGGCAGATCC | TGAAGTCATCAGCTGCCAGG |
| S100A7 | AGCCATACTACATCACAGA | TACAGGAACTCATCAAAGC |
| β-actin | GGCTGTATTCCCCTCCATCG | CCAGTTGGTAACAATGCCATGT |
| Muc-2 | GCTGACGAGTGGTTGGTGAATG | GATGAGGTGGCAGACAGGAGAC |
| *Parabacteroides distasonis* | CATTCGGACCGAGGTGGAAA | CCACCAGAGTCCTCAGCTTTA |
| *Prevotella* | CCAGCCAAGTAGCGTGCA | TGGACCTTCCGTATTACCGC |
| *Bacteria* | ACTCCTACGGGAGGCAGCAG | ATTACCGCGGCTGCTGG |
| **Probes** | **Sequence and fluorophore** | |
| *Parabacteroides distasonis* | 5′ FAM- CAGCGATGAATCTTTAGCAAATATCC | |
| *Prevotella* | 5′ Texas Red-GCACGCTACTTGGCTGG | |
